# GOLD COPD Exacerbation History Categories and Disease Outcomes

**DOI:** 10.1001/jamanetworkopen.2024.45488

**Published:** 2024-12-18

**Authors:** Kiki Waeijen-Smit, Daphne E. M. Peerlings, Rudolf A. Jörres, Henrik Watz, Robert Bals, Klaus F. Rabe, Claus F. Vogelmeier, Tim Speicher, Martijn A. Spruit, Sami O. Simons, Sarah Houben-Wilke, Frits M. E. Franssen, Peter Alter

**Affiliations:** 1 Department of Research and Development, Ciro, Horn, the Netherlands; 2 Department of Respiratory Medicine, Research Institute of Nutrition and Translational Research in Metabolism, Faculty of Health Medicine and Life Sciences, Maastricht University Medical Centre+, Maastricht, the Netherlands; 3 Institute and Outpatient Clinic for Occupational, Social and Environmental Medicine, Munich, Germany; 4 LungenClinic Grosshansdorf, Airway Research Center North, German Center for Lung Research, Grosshansdorf, Germany; 5 Velocity Clinical Research Grosshansdorf, Grosshansdorf, Germany; 6 Department of Internal Medicine V-Pulmonology, Allergology, Critical Care Medicine, Saarland University Hospital, Homburg, Germany; 7 Helmholtz Institute for Pharmaceutical Research Saarland, Helmholtz Centre for Infection Research, Saarland University Campus, Saarbrücken, Germany; 8 Department of Medicine, Christian Albrechts University Kiel, Kiel and LungenClinic Grosshansdorf (Members of the German Center for Lung Research), Grosshansdorf, Germany; 9 Department of Medicine, Pulmonary, Critical Care and Sleep Medicine, Philipps-University of Marburg, Member of the German Center for Lung Research, Marburg, Germany

## Abstract

**Importance:**

Previous exacerbations of chronic obstructive pulmonary disease (ECOPD) are associated with future events. For more than a decade, patients at high risk have been defined as individuals with a history of 2 or more moderate ECOPD, 1 or more severe ECOPD, or both within 12 months, and treatments have been allocated accordingly, but these cutoffs lack validation.

**Objectives:**

To validate ECOPD history categories by the Global Initiative for Chronic Obstructive Lung Disease (GOLD) and explore alternative cutoffs to estimate moderate and severe ECOPD and all-cause mortality in COPD.

**Design, Setting, and Participants:**

This cohort study analyzed data from patients with COPD in the German COPD and Systemic Consequences-Comorbidities Network (COSYCONET) study. Patients were recruited from September 2010 to December 2013. Analyses were conducted in September 2023 to August 2024.

**Main Outcomes and Measures:**

Risk of moderate and severe (ie, with hospitalization) ECOPD and all-cause mortality over a 4.5-year follow-up period were assessed using binomial logistic regressions and area under the receiver operating characteristic curves (AUROCs) with 95% CIs.

**Results:**

Among 2291 patients with COPD GOLD categories 1 to 4 (mean [SD] age, 65 [8] years; 1396 male [60.9%]), the mean (SD) estimated forced expiratory volume in the first second of expiration was 52.5% (18.6%). ECOPD history categories by GOLD had an AUROC of 0.63 (95% CI, 0.60-0.65) and 0.62 (95% CI, 0.58-0.66) to estimate moderate and severe ECOPD, respectively. A single previous moderate ECOPD within 12 months more accurately estimated future moderate and severe ECOPD (AUROC, 0.66; 95% CI, 0.64-0.69), and in line with GOLD, 1 previous severe ECOPD within 12 months estimated moderate and severe ECOPD (AUROC, 0.63; 95% CI, 0.60-0.67). The 4-year mortality rate was 219 patients (9.6%). Patients with 3 or more previous moderate ECOPD (odds ratio, 2.18; 95% CI, 1.27-3.72) or 1 or more previous severe ECOPD (odds ratio, 1.57; 95% CI, 1.29-1.91) within 12 months were more likely to die compared with patients without prior ECOPD.

**Conclusions and Relevance:**

This study’s findings suggest a limited estimative performance of ECOPD history categories by GOLD. Novel cutoffs were suggested, categorizing patients as without exacerbations or with high-risk exacerbations based on a history of 1 or more moderate ECOPD, 1 or more severe ECOPD, or both within 12 months.

## Key Points

**Question:**

How accurate are exacerbations of chronic obstructive pulmonary disease (ECOPD) history categories by the Global Initiative for Chronic Obstructive Lung Disease (GOLD) in estimating moderate and severe ECOPD and all-cause mortality in patients with COPD?

**Findings:**

In this cohort study of 2291 German patients with COPD, GOLD ECOPD history categories had an area under the receiver operating characteristic curve of 0.63 for moderate and 0.62 for severe ECOPD. While alternative cutoffs were associated with improved estimation, model performance remained mediocre.

**Meaning:**

These findings suggest that GOLD ECOPD history categories may be limited in estimating future COPD outcomes but that lowering the threshold for moderate ECOPD may be associated with improved performance.

## Teaser

This cohort study investigates the accuracy of Global Initiative for Chronic Obstructive Lung Disease (GOLD) exacerbations of COPD history categories in estimating COPD disease outcomes in German patients.

## Introduction

Patients with chronic obstructive pulmonary disease (COPD) are at increased risk of experiencing sudden deteriorations in respiratory health, called exacerbations of COPD (ECOPD). Approximately 30% of patients experience frequent ECOPD,^1^ defined as 2 or more annual ECOPD.^2^ Recognition of the pivotal role of ECOPD in the course of COPD led to the development of the ABCD assessment tool in the 2011 Global Initiative for Chronic Obstructive Lung Disease (GOLD) strategy document, the international clinical standard for the diagnosis, treatment, and prevention of COPD.^3^ Disease classification was no longer strictly reflected by the degree of airflow limitation, but also by ECOPD history and symptoms. In 2023, GOLD proposed a further evolution of the ABCD assessment tool, recognizing the clinical relevance of ECOPD independently of the level of symptoms (GOLD ABE assessment tool).^4^

In both assessment tools, ECOPD history is divided into moderate ECOPD (ie, events treated with bronchodilators and oral corticosteroids, antibiotics, or both) and severe events (ie, those requiring hospitalization or a visit to the emergency department). Risk of future ECOPD is estimated based on the frequency of previous ECOPD (<12 months): 2 or more moderate ECOPD indicate high risk, whereas 1 or fewer moderate ECOPD indicate low risk.^2,3^ These cutoffs are based on previous research showing that the most important determinant of ECOPD is a history of frequent ECOPD.^1^ Furthermore, hospitalization for ECOPD also indicates high ECOPD risk.^2,3,5^ Patients at high ECOPD risk are considered to be in GOLD group E, whereas patients at low ECOPD risk are considered to be in GOLD group A or B depending on their symptom burden.^2^ Assessment of ECOPD risk according to the GOLD classification system holds a central place in the clinical management of COPD given that this assessment is used to drive therapeutic recommendations and estimate COPD and other outcomes.^2^ Therefore, accurate ECOPD risk assessment is essential.

An increasing number of studies have shown that a history of 2 or more moderate ECOPD may not be on a par with prior severe ECOPD in terms of ECOPD risk and all-cause mortality. Indeed, little difference was observed in the risk of hospitalization or mortality between patients in GOLD groups B and C.^6^ However, the definition of the groups may have played a role given that it is dependent on the definition of symptoms used.^7,8,9^ A 2022 study^10^ in patients in GOLD group B with 1 previous moderate ECOPD found that the odds of experiencing 2 or more subsequent moderate ECOPD were higher than the odds of experiencing 1 or more severe ECOPD during a 3-year follow-up. In contrast, the risk of future ECOPD was higher in patients with 1 or more severe ECOPD compared with patients with 2 or more previous moderate ECOPD in a general practice–based population.^11^ Furthermore, the risk of severe but not moderate ECOPD was substantially higher in patients with a hospitalization history compared with patients with previous moderate ECOPD.^12^ Altogether, contrasting results have been reported.

Therefore, this study aimed to evaluate the estimating performance of the current ECOPD history categories by GOLD using data of the COPD and Systemic Consequences-Comorbidities Network (COSYCONET) cohort.^13^ More specifically, we investigated whether there was a difference in the risk of 1- and 4-year moderate and severe ECOPD and 4-year all-cause mortality based on the occurrence of 2 or more moderate ECOPD vs 1 or more severe ECOPD in the previous year and explored potential superior cutoffs of ECOPD history for the estimation of moderate and severe ECOPD and all-cause mortality.

## Methods

### Study Population and Measurements

This cohort study was a post hoc analysis using data from patients with COPD who participated in the clinical German COSYCONET study. The detailed study protocol has been described elsewhere.^13^ This study followed the Strengthening the Reporting of Observational Studies in Epidemiology (STROBE) reporting guideline for cohort studies. COSYCONET is registered at ClinicalTrials.gov (NCT01245933). COSYCONET complied with the Declaration of Helsinki and Good Clinical Practice Guidelines and has been approved by the ethics committees of the participating centers and the concerned data security authority. All participants provided written informed consent.^13^ The data used in this study were existing and deidentified, and therefore, the study was deemed exempt from further institutional review board review or informed consent requirements under 45 CFR §46.104(d)(4) of the Common Rule, which allows exemption for research involving secondary analysis of existing, nonidentifiable data.

In COSYCONET, ECOPD were defined as acute and significant worsenings of lung disease (eg, increased shortness of breath or increased or purulent sputum) that lasted several days and required taking special measures. This definition resonates with the definition provided by GOLD, and for the purpose of this study, these events were referred to as moderate ECOPD. In COSYCONET, severe ECOPD were defined as hospital admissions, excluding visits to the emergency department. For the purpose of this study, these events were referred to as severe ECOPD. Assessments took place at 6, 18, 36, and 54 months (visits 2, 3, 4, and 5 [V2-5], respectively) after the baseline visit (visit 1 [V1]) (eFigure 1 in Supplement 1). Further details on these assessments and other measurements included in this study are provided in the eMethods and eResults in Supplement 1.

### Statistical Analysis

Baseline characteristics are presented as frequencies and percentages, means and SDs, or medians and IQRs, as appropriate. The Shapiro-Wilk test and visual inspection of normality plots were performed to test for normality. Moderate and severe ECOPD trajectories are visualized with Sankey diagrams produced using the RStudio Integrated Development for R software version 4.3.1 (RStudio) (eMethods in Supplement 1).

To estimate moderate and severe ECOPD and all-cause mortality risk based on ECOPD history (<12 months), binomial logistic regressions and area under the receiver operating characteristic curves (AUROCs) were constructed. Separate (ie, nonrepeated) analyses were performed among all patients with available data: 1- and 4-year ECOPD risk status estimates were calculated using V2 and V3 and V2 and V5, respectively, whereas 4-year all-cause mortality risk was calculated using mortality status at V5. With respect to ECOPD status at V3 and V5, moderate and severe ECOPD were separately entered into models. Binomial logistic regressions were performed to construct crude and adjusted odds ratios (ORs) with 95% CIs. Dependent variables were ECOPD status (yes or no) at V3 and V5 and mortality status at V5 (yes or no), while the independent variable was ECOPD history at V2 (categorized as 0, 1, 2, 3, and ≥4 ECOPD). Adjustments for age (years), sex (male or female), smoking status (never, current, or former smoker), forced expiratory volume in the first second of expiration (FEV_1_; estimated percentage), and cardiac comorbidities (ie, heart failure, cardiac arrhythmia, or previous cardiac arrest; yes or no) were performed given their strong association with ECOPD risk.^2,14^

AUROCs with 95% CIs and precision recall curves were constructed to study the estimating performance of the current GOLD model and to study the proposed model with potentially superior cutoff values, again encompassing all patients with available data. Dependent variables were ECOPD status (yes or no) at V3 and V5, while the independent variable consisted of ECOPD categories at V2 categorized according to the current GOLD model and the proposed model (yes or no for ECOPD history). The Youden index was used to select the optimal cutoff point.^15^ Precision recall AUROC curves were plotted in Python programming language version 3.12.4 (Python Software Foundation) using the package scikit-learn version 1.5.1. A logistic regression with default parameters was used to obtain precision and recall values. No test or train split was made.^16^ Current GOLD and novel AUROC curves were compared using a paired-sample design. A priori, 2-sided *P* values ≤.05 were considered statistically significant. Statistical analyses were conducted using SPSS statistical software version 28.0.1.1 (IBM). Analyses were conducted in September 2023 to August 2024.

## Results

Among 2291 patients (mean [SD] age, 65 [8] years; 1396 male [60.9%]) included in the study (eFigure 1 in Supplement 1; Table 1), the mean (SD) estimated FEV_1_ was 52.5% (18.6%). Analyses were performed cross-sectionally, including all patients with available data at V2 and V3 for 1-year risk estimations, corresponding to 1687 and 1688 included patients with moderate and severe ECOPD, respectively, and at V2 and V5 for 4-year risk estimations, corresponding to 861 and 856 included patients with moderate and severe ECOPD, respectively. Almost one-quarter of patients had preexisting cardiac disease (282 of 1192 patients with data [23.7%]). During the 4.5-year study follow-up period, 219 patients (9.6%) died. Among 853 patients initially in GOLD group E, 42 patients (4.9%) remained in group E over the course of study follow-up. Baseline patient characteristics of 828 patients (36.1%) who had no missing ECOPD data during study follow-up (ie, throughout V1-V5) are presented in eTable 1 in Supplement 1. Please see eFigure 2 in Supplement 1 for ECOPD trajectories of these patients.

**Table 1.** Baseline Study Population Characteristics

Characteristic	Patients, No. (N = 2291)				
	Visit 1 (n = 2291)	Visit 2 (n = 1996)	Visit 3 (n = 1724)	Visit 4 (n = 1182)	Visit 5 (n = 878)
Sex					
Male	1396 (60.9)	1230 (61.6)	1046 (60.7)	719 (60.8)	519 (59.1)
Female	895 (39.1)	766 (38.4)	678 (39.3)	463 (39.2)	359 (40.9)
Age, mean (SD), y	65.1 (8.4)	65.5 (8.3)	66.3 (8.2)	67.4 (8.2)	68.3 (8.2)
Years since diagnosis					
Median (IQR)	6.0 (3.0-10.0)	NA	NA	NA	NA
Patients with data, No.	2271	NA	NA	NA	NA
GOLD group					
A	247 (10.8)	279 (14.1)	202 (11.8)	148 (12.6)	108 (12.5)
B	1205 (52.9)	1167 (58.8)	990 (57.7)	657 (55.7)	491 (56.6)
E	827 (36.3)	539 (27.2)	523 (30.5)	374 (31.7)	268 (30.9)
Patients with data, No.	2279	1985	1715	1179	867
Lung function tests					
Estimated FEV_1_					
Mean (SD), %	52.5 (18.6)	53.1 (18.7)	52.3 (18.8)	53.0 (19.2)	53.6 (19.7)
Patients with data, No.	2291	1985	1704	1177	878
Estimated FVC					
Mean (SD), %	78.2 (19.2)	78.4 (18.8)	77.9 (19.3)	78.2 (19.6)	79.5 (19.9)
Patients with data, No.	2291	1985	1704	1176	862
Estimated TLCO					
Mean (SD), %	52.6 (20.7)	52.5 (20.4)	52.6 (20.7)	53.9 (21.7)	54.2 (20.5)
Patients with data, No.	2162	1890	1620	1118	823
Smoking status					
Current smoker	563 (24.6)	447 (22.4)	336 (19.5)	207 (17.5)	149 (17.0)
Former smoker	1574 (68.8)	1412 (70.9)	1259 (73.2)	888 (75.2)	658 (75.3)
Never smoker	150 (6.6)	133 (6.7)	125 (7.3)	86 (7.3)	67 (7.7)
Patients with data, No.	2287	1992	1720	1181	874
Pack-years					
Median (IQR)	41.0 (19.0-63.8)	41.0 (18.6-64.5)	41.7 (19.6-64.5)	42.0 (19.0-64.5)	42.0 (19.5-64.5)
Patients with data, No.	2277				
Medications					
LABA	104 (4.5)	86 (4.3)	66 (3.8)	46 (3.9)	28 (3.2)
LAMA	151 (6.6)	144 (7.2)	121 (7.0)	64 (5.4)	58 (6.6)
ICS	15 (0.7)	15 (0.8)	15 (0.9)	18 (1.5)	8 (0.9)
LABA and LAMA	355 (15.5)	296 (14.8)	256 (14.8)	215 (18.2)	175 (19.9)
ICS and LAMA	24 (1.0)	28 (1.4)	22 (1.3)	11 (0.9)	8 (0.9)
ICS and LABA	277 (12.1)	217 (10.9)	199 (11.5)	119 (10.1)	79 (9.0)
ICS, LABA, and LAMA	1192 (52.0)	1019 (51.1)	835 (48.4)	547 (46.3)	402 (45.8)
Oxygen use	444 (19.4)	286 (16.9)	284 (19.5)	200 (20.0)	138 (18.6)
Patients with data, No.	2290	1694	1458	1002	741
mMRC ≥2	1097 (48.3)	939 (47.4)	789 (45.9)	567 (48.2)	420 (48.3)
Patients with data, No.	2270	1981	1718	1177	870
CAT, total score, mean (SD)	18.0 (7.0)	18.0 (8.0)	18.0 (7.0)	18.0 (7.0)	18.0 (8.0)
Patients with data, No.	2276	1986	1718	1176	874
ECOPD <12 mo^a^					
Moderate, No.					
0	1037 (45.3)	1082 (54.3)	893 (51.9)	608 (51.4)	452 (51.7)
1	611 (26.7)	483 (24.2)	405 (23.5)	268 (22.7)	204 (23.3)
2	278 (12.1)	189 (9.5)	194 (11.3)	134 (11.3)	104 (11.9)
3	154 (6.7)	113 (5.7)	102 (5.9)	86 (7.3)	55 (6.3)
≥4	210 (9.2)	127 (6.4)	126 (7.3)	86 (7.3)	59 (6.8)
Patients with data, No.	2290	1994	1720	1182	874
Severe, No.					
0	1830 (79.9)	1747 (87.6)	1475 (85.7)	1014 (85.8)	737 (84.8)
1	320 (14.0)	179 (9.0)	168 (9.8)	108 (9.1)	85 (9.8)
2	87 (3.8)	39 (2.0)	51 (3.0)	34 (2.9)	30 (3.5)
3	32 (1.4)	18 (0.9)	16 (0.9)	13 (1.1)	9 (1.0)
≥4	20 (0.9)	11 (0.6)	11 (0.6)	13 (1.1)	8 (0.9)
Patients with data, No.	2289	1994	1721	1182	869
Cardiac comorbidities					
Heart failure	117 (9.8)	122 (8.5)	143 (8.4)	104 (8.8)	95 (10.8)
Patients with data, No.	1192	1437	1702	1182	877
Cardiac arrhythmia	194 (16.3)	199 (13.9)	241 (14.2)	190 (16.1)	162 (18.5)
Patients with data, No.	1193	1436	1703	1182	877
Cardiac arrest	98 (4.3)	85 (4.3)	85 (4.9)	61 (5.2)	60 (6.8)
Patients with data, No.	2291	1994	1723	1182	876

Abbreviations: CAT, COPD Assessment Test; ECOPD, exacerbations of chronic obstructive pulmonary disease; FEV_1_, forced expiratory volume in the first second of expiration; FVC, forced vital capacity; GOLD, Global Initiative for Chronic Obstructive Lung Disease; ICS, inhaled corticosteroid; LABA, long-acting beta-agonist; LAMA, long-acting muscarinic antagonist; mMRC, modified Medical Research Council; NA, not applicable; TLCO, transfer factor for carbon monoxide.

^a^ ECOPD history within 12 months was recorded at each study visit; values presented concern the respective history per study visit.

### ECOPD History and Future Moderate ECOPD

The AUROC for the current ECOPD history categories for 1-year moderate ECOPD risk estimation was 0.63 (95% CI, 0.60-0.65), with a sensitivity of 39.8% (95% CI, 36.4%-43.2%) and a specificity of 85.5% (95% CI, 83.2%-87.8%) (eFigure 3 in Supplement 1). The precision recall AUROC was 0.59 (eFigure 4 in Supplement 1). A similar AUROC curve was observed for 4-year moderate ECOPD risk estimation; the AUROC was 0.60 (95% CI, 0.56-0.64), with a sensitivity of 35.1% (95% CI, 30.5%-39.7%) and a specificity of 85.3% (95% CI, 82.0%-88.6%) (eFigure 3 in Supplement 1). The precision recall AUROC was 0.56 (eFigure 4 in Supplement 1).

The cutoff value with the optimal sensitivity and specificity scores to estimate 1-year moderate ECOPD was 1 moderate and 1 severe ECOPD event within 12 months (eTable 2 in Supplement 1). Similar to 1-year moderate ECOPD risk estimation, a cutoff of 1 moderate ECOPD and 1 severe ECOPD event within 12 months showed the optimal sensitivity and specificity scores to estimate 4-year moderate ECOPD risk (eTable 3 in Supplement 1).

Patients with previous moderate ECOPD were more likely to experience future moderate ECOPD compared with patients without prior events at V3 (OR, 1.84; 95% CI, 1.66-2.03; Nagelkerke *R*^2^, 13.4 [crude] and 17.0 [adjusted]) and V5 (OR, 1.68; 95% CI, 1.47-1.92; Nagelkerke *R*^2^, 10.1 [crude] and 21.4 [adjusted]). Similar ORs were observed for a severe ECOPD history (V3: OR, 1.96; 95% CI, 1.56-2.45; Nagelkerke *R*^2^, 3.3 [crude] and 10.2 [adjusted]; V5: OR, 2.00; 95% CI, 1.39-2.86; Nagelkerke *R*^2^, Nagelkerke R2: 2.7 [crude] and 15.5 [adjusted]) (Table 2). Adjusted ORs were reduced but still statistically significant. See an overview of the contribution of covariates in the adjusted values for moderate ECOPD risk (1- and 4-year estimation) in eTable 4 in Supplement 1.

**Table 2.** Association of ECOPD History at V2 With Odds of Moderate ECOPD Status

ECOPD history <12 mo	Moderate ECOPD							
	1 y				4 y			
	Crude, OR (95% CI)^a^	*P* value	Adjusted, OR (95% CI)^a^	*P* value	Crude, OR (95% CI)^a^	*P* value	Adjusted, OR (95% CI)^a^	*P* value
Moderate								
0	1 [Reference]	NA	1 [Reference]	NA	1 [Reference]	NA	1 [Reference]	NA
≥1	1.84 (1.66-2.03)	<.001	1.71 (1.52-1.92)	<.001	1.68 (1.47-1.92)	<.001	1.65 (1.40-1.95)	<.001
χ^2^ (*df*)	178.78 (1)	<.001	164.94 (8)	<.001	67.48 (1)	<.001	108.53 (8)	<.001
Nagelkerke *R*^2^	13.4	NA	17.0	NA	10.1	NA	21.4	NA
Patients included in the analysis, No. (%)	1687 (73.6)	NA	1210 (52.8)	NA	861 (37.6)	NA	621 (27.1)	NA
Severe								
0	1 [Reference]	NA	1 [Reference]	NA	1 [Reference]	NA	1 [Reference]	NA
≥1	1.96 (1.56-2.45)	<.001	1.84 (1.42-2.39)	<.001	2.00 (1.39-2.86)	<.001	1.77 (1.18-2.65)	.006
χ^2^ (*df*)	42.38 (1)	<.001	96.43 (8)	<.001	17.50 (1)	<.001	76.80 (8)	<.001
Nagelkerke R^2^	3.3	NA	10.2	NA	2.7	NA	15.5	NA
Patients included in the analysis, No. (%)	1687 (73.6)	NA	1210 (52.8)	NA	861 (37.6)	NA	621 (27.1)	NA

Abbreviations: *df*, degrees of freedom; ECOPD, exacerbations of chronic obstructive pulmonary disease; NA, not applicable; V, visit.

^a^ ORs are shown for moderate ECOPD status at 1 and 4 years by history of ECOPD at V2. Adjustment was performed for age, sex, smoking status, estimated forced expiratory volume in the first second of expiration, and cardiac comorbidities (ie, heart failure, cardiac arrhythmia, and cardiac arrest) at baseline.

### ECOPD History and Future Severe ECOPD

The AUROC for current ECOPD history categories for 1-year estimation of severe ECOPD was 0.62 (95% CI, 0.58-0.66), with a sensitivity of 47.3% (95% CI, 74.6%-53.6%) and a specificity of 76.8% (95% CI, 74.6%-79.0%) (eFigure 5 in Supplement 1). The precision recall AUROC was 0.46 (eFigure 6 in Supplement 1). A similar AUROC curve was observed for 4-year severe ECOPD risk estimation; the AUROC was 0.61 (95% CI, 0.55-0.66), with a sensitivity of 42.7% (95% CI, 34.2%-51.2%) and a specificity of 78.8% (95% CI, 75.8%-81.8%) (eFigure 5 in Supplement 1). The precision recall AUROC was 0.44 (eFigure 6 in Supplement 1).

For 1- and 4-year severe ECOPD estimation, the cutoff with the optimal sensitivity and specificity was 1 moderate ECOPD event (sensitivity: 67.9%; 95% CI, 62.0%-73.8% and 59.5%; 95% CI, 51.1%-67.9%, respectively; specificity: 58.2%; 95% CI, 55.7%-60.7% and 59.6%; 95% CI, 56.0%-63.2%, respectively) and 1 severe ECOPD event (sensitivity: 31.2%; 95% CI, 25.3%-37.1% and 26.0%; 95% CI, 18.5%-33.5%, respectively; specificity: 91.6%; 95% CI, 90.2%-93.0% and 93.4%; 95% CI, 91.6%-95.2%, respectively) within 12 months. AUROCs of these models were comparable at 1- and 4-year estimation (eTables 6 and 7 in Supplement 1).

Compared with patients without prior events, patients with previous moderate ECOPD were more likely to experience severe ECOPD at V3 (OR, 1.36; 95% CI, 1.23-1.51) and V5 (OR, 1.36; 95% CI, 1.18-1.56), as were patients with previous severe ECOPD (V3: OR, 2.29; 95% CI, 1.87-2.81; V5: 2.61; 95% CI, 1.87-3.65). Adjusted OR values were reduced. See an overview of the contribution of covariates in the adjusted values for severe ECOPD risk (1- and 4-year estimation) in eTable 5 in Supplement 1. All model outcomes were statistically significant but explained little variance of the model (Table 3).

**Table 3.** Association of ECOPD History at V2 With Odds of Severe ECOPD Status

ECOPD history <12 mo	Severe ECOPD							
	V3				V5			
	Crude, OR (95% CI)^a^	*P* value	Adjusted, OR (95% CI)^a^	*P* value	Crude, OR (95% CI)^a^	*P* value	Adjusted, OR (95% CI)^a^	*P* value
Moderate								
0	1 [Reference]	NA	1 [Reference]	NA	1 [Reference]	NA	1 [Reference]	NA
≥1	1.36 (1.23-1.51)	<.001	1.28 (1.13-1.46)	<.001	1.36 (1.18-1.56)	<.001	1.29 (1.08-1.55)	<.001
χ^2^ (*df*)	32.89 (1)	<.001	78.29 (8)	<.001	16.81 (1)	<.001	60.75 (8)	<.001
Nagelkerke *R*^2^	3.5	NA	11.2	NA	3.4	NA	16.2	NA
Patients included in the analysis, No. (%)	1688 (73.7)	NA	1211 (52.9)	NA	856 (37.4)	NA	616 (26.9)	NA
Severe								
0	1 [Reference]	NA	1 [Reference]	NA	1 [Reference]	NA	1 [Reference]	NA
≥1	2.29 (1.87-2.81)	<.001	2.30 (1.80-2.94)	<.001	2.61 (1.87-3.65)	<.001	2.35 (0.42-4.79)	<.001
χ^2^ (*df*)	64.48 (1)	<.001	110.06 (8)	<.001	33.12 (1)	<.001	70.54 (8)	<.001
Nagelkerke *R*^2^	6.7	NA	15.6	NA	6.6	NA	18.6	NA
Patients included in the analysis, No. (%)	1688 (73.7)	NA	1211 (52.9)	NA	856 (37.4)	NA	616 (26.9)	NA

Abbreviation: *df*, degrees of freedom; ECOPD, exacerbations of chronic obstructive pulmonary disease; NA, not applicable; V, visit.

^a^ ORs are shown for severe ECOPD status at V3 (18 months) and V5 (54 months) by history of ECOPD at V2 (6 months). Adjustment was performed for age, sex, smoking status, estimated forced expiratory volume in the first second of expiration, and cardiac comorbidities (ie, heart failure, cardiac arrhythmia, and cardiac arrest).

### ECOPD History and All-Cause Mortality

The AUROC for the current ECOPD history categories for 4-year all-cause mortality estimation was 0.55 (95% CI, 0.51-0.60), with a sensitivity of 36.6% (95% CI, 29.6%-43.6%) and a specificity of 73.7% (95% CI, 71.7%-75.7%) (eFigure 7 in Supplement 1). The precision recall AUROC was 0.36 (eFigure 8 in Supplement 1).

Curves of moderate and severe ECOPD history for all-cause mortality are depicted in eTable 8 in Supplement 1. The cutoff with the optimal sensitivity and specificity to estimate 4-year all-cause mortality was 3 moderate ECOPD and 1 severe ECOPD event within 12 months.

Odds for 4-year all-cause mortality were similar for patients with 1 or 2 previous moderate ECOPD compared with patients without previous moderate ECOPD. Patients with 3 or more moderate ECOPD were more likely to die within 4 years compared with patients without previous ECOPD (OR, 2.18; 95% CI, 1.27-3.72). Patients with previous severe ECOPD were more likely to die within 4 years compared with patients without previous severe ECOPD (OR, 1.57; 95% CI, 1.29-1.91) (eTable 9 in Supplement 1). See an overview of the contribution of covariates in the adjusted values for all-cause mortality in eTable 10 in Supplement 1.

### Proposal for Novel ECOPD History Grading

Based on these findings, a novel grading system was constructed based on 1 moderate ECOPD event, 1 severe ECOPD event, or both within 12 months (Figure). The inference about the area difference under ROC curves is summarized in eTable 11 in Supplement 1. For 1- and 4-year moderate ECOPD risk estimation, the AUROC of the proposed grading cutoffs was 0.66 (95% CI, 0.64, 0.69) and 0.62 (95% CI, 0.58, 0.66), respectively, with a sensitivity of 62.6% (95% CI, 59.3%-66.0%) and 55.7% (95% CI, 50.9%-60.4%) and a specificity of 70.1% (95% CI, 67.1%-73.1%) and 67.9% (95% CI, 63.4%-72.0%), respectively (Figure, A and C). The precision recall AUROC was 0.67 for 4-year ECOPD and 0.72 for 1-year ECOPD (eFigure 9 in Supplement 1). This represents a substantial improvement from the current GOLD criteria for 1-year moderate ECOPD estimation (eTable 11 in Supplement 1). For 1- and 4-year severe ECOPD risk estimation, the AUROC of the proposed grading cutoffs was 0.63 (95% CI, 0.60-0.67) and 0.60 (95% CI, 0.54-0.65), respectively, with a sensitivity of 68.4% (95% CI, 62.5%-74.3%) and 59.5% (95% CI, 51.1%-67.9%), respectively, and a specificity of 58.2% (95% CI, 55.7%-60.7%) and 59.4% (95% CI, 55.8%-63.0%), respectively (Figure, B and D). The precision recall AUROC was 0.63 for 1-year and 0.57 for 4-year estimation (eFigure 9 in Supplement 1). The latter models were not substantially different from current GOLD criteria (eTable 11 in Supplement 1).

**Figure.**
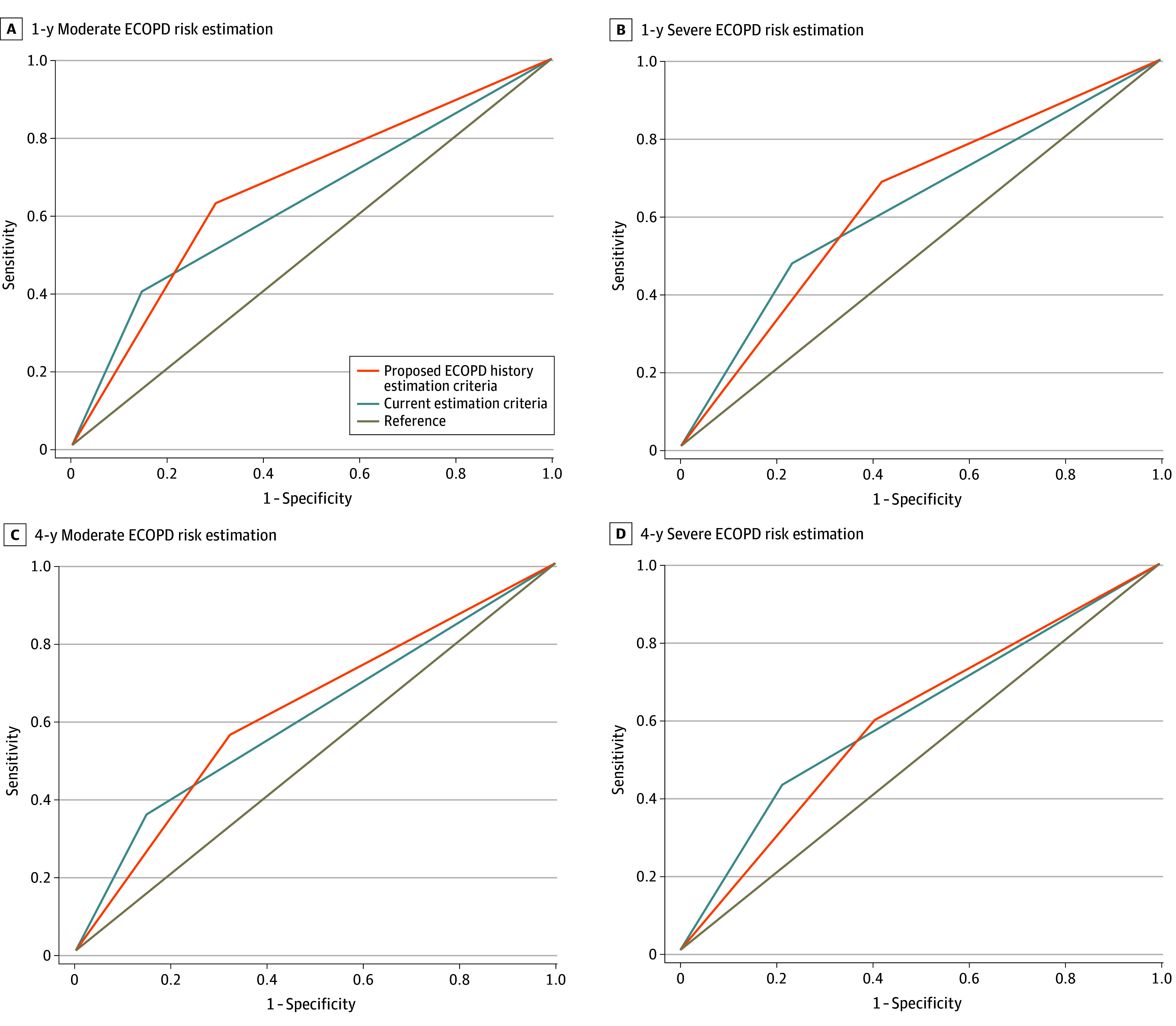
Exacerbations of Chronic Obstructive Pulmonary Disease (ECOPD) Risk Estimation The figure shows area under the receiver operating characteristic curves of the proposed ECOPD history categories vs the current criteria by the Global Initiative for Chronic Obstructive Lung Disease (GOLD) to estimate 1-year (visit 2 vs visit 3) moderate ECOPD risk among 1687 patients (A), 1-year severe ECOPD risk among 1688 patients (B), 4-year (visit 2 vs visit 5) moderate ECOPD risk among 861 patients (C), and 4-year severe ECOPD risk among 856 patients (D).

## Discussion

This study revealed poor performance of GOLD’s current cutoffs of 2 or more moderate ECOPD, 1 or more severe ECOPD, or both for estimating future COPD-related outcomes. The risk of future moderate or severe ECOPD and all-cause mortality was similar over a 54-month follow-up period between patients with a moderate and severe ECOPD history. A history of 1 moderate ECOPD event, 1 severe ECOPD event, or both was associated with improved performance for GOLD’s current model regarding short-term (1-year) and long-term (4-year) risk estimation for moderate and severe ECOPD. However, models still had a mediocre performance, suggesting that ECOPD risk cannot be accurately estimated by ECOPD history alone. Despite this finding, ECOPD history remains important given that the inclusion of additional factors did not abolish its estimative value. Lowering the cutoff for moderate ECOPD resonates with the current COPD paradigm that each single ECOPD event matters.^17,18^ When validated by other cohorts, the current findings may mean a significant shift in COPD treatment initiation or escalation.

Since 2011, high future ECOPD risk has been stratified according to a history of 2 or more moderate ECOPD, 1 or more severe ECOPD, or both.^19^ In line with other studies,^6,10,11,12^ this study’s findings suggest that these criteria may not be accurate for risk estimation in COPD. In fact, GOLD’s current model poorly estimated 1- and 4-year moderate and severe ECOPD risk. Hence, novel cutoff values were explored and were associated with substantially improved sensitivity. The proposed grading system based on 1 or more moderate ECOPD, 1 or more severe ECOPD, or both presented a small but significant improvement over GOLD’s current criteria in terms of moderate ECOPD risk estimation and could be easily implemented using existing data. Importantly, although a small significant difference in the pairwise area was observed, the justification for altering this widely used metric, as well as the clinical relevance of the metric, need to be carefully evaluated in future studies.

Our findings are supported by others, including a 2023 study^20^ that demonstrated that a history of 1 ECOPD event was associated with a high risk of moderate and severe ECOPD in patients in GOLD groups A and B. As such, these results may support a shift away from differentiating between moderate or severe ECOPD history to categorizing patients as having no exacerbations or with high-risk exacerbations. Given that the difference between moderate and severe ECOPD is traditionally based on the intensity of health care use, this new categorization may be considered more objective and transparent. Additionally, it may transform patient treatment, which is not only relevant for individuals living with COPD but also for their caregivers and clinicians, health insurance companies, researchers, policymakers, and lung health organizations and patient associations. Importantly, given that recommendations for pharmacotherapy for COPD are based on GOLD ABE groups, the impact of a lower ECOPD threshold on individual patient treatment, potential overtreatment, and related financial implications should be evaluated in future studies.

In this study, 52.0% of patients were taking triple pharmacotherapy at the baseline visit. Over the years, increased attention has been paid to the prescription of triple pharmacotherapy,^2^ while positive outcomes in frequency and severity of ECOPD and all-cause mortality have been shown in patients who were symptomatic with a history of ECOPD in previous studies.^21,22^ Therefore, validation of the current proposed criteria in an external, more recent cohort of patients with COPD is needed.

Importantly, the discriminative accuracy of the proposed models remained limited (ie, AUROC <0.70). This stresses the need to further evaluate the general adequacy of the ABE model. Previous research revealed that cardiovascular comorbidities in particular were associated with frequent ECOPD.^23^ Vice versa, cardiovascular events are common after ECOPD.^17,24,25^ Indeed, the vast majority of patients with COPD have multiple comorbidities, including hyperglycemia, atherosclerosis, and hypertension.^23,26^ In this study, almost one-quarter of patients had preexisting cardiac disease (282 of 1192 patients with data [23.7%]). Lowering the threshold to 1 moderate ECOPD event should also be seen in the context of this post-ECOPD increased cardiopulmonary risk.^27^ A 2024 study^28^ found that moderate ECOPD was associated with an increased risk of major cardiovascular events, and a 2024 study^17^ found that this risk occurred even in newly diagnosed individuals experiencing their first ever ECOPD. This once again emphasizes the importance of ECOPD prevention.^18^

As supported by other studies,^12,29,30^ we found that ECOPD risk was similar regardless of severity but rather was associated with the presence of previous events, whereas risk of severe events was substantially higher for patients with a more severe ECOPD history. Aside from established risk factors associated with ECOPD, including age, sex, smoking status, and FEV_1_, cardiac comorbidities were also included as covariates provided their potential association with ECOPD.^31,32,33^ Of all included covariates and as a previous study^34^ found, FEV_1_ was consistently associated with improved estimation of moderate and severe ECOPD. Consistent with this, GOLD’s 2007 classification strictly based on FEV_1_^19^ revealed a better discriminative accuracy for estimating severe ECOPD and all-cause mortality than GOLD 2011.^35^ We found that cardiac arrhythmia had the best performance for estimating 4-year moderate ECOPD risk, whereas heart failure added significantly to the estimation of 4-year all-cause mortality risk. While the estimative ability of the different models studied improved substantially in adjusted models, the explained variance remained low. It is noteworthy that the proportion of patients with cardiac comorbidities was small, limiting the power to detect associations of these conditions with ECOPD. Hence, future studies should further explore outcomes associated with such comorbidities and other factors associated with ECOPD risk. Disease severity, comorbidities, symptom burden, and blood eosinophil counts were recently identified as important factors aside from ECOPD history associated with ECOPD.^36^ Many of these factors were similarly identified in patients with COPD in GOLD groups A or B.^37^ How these factors may be integrated in COPD risk assessment classification tools remains to be elucidated.

In total, 9.6% of patients died during follow-up in our study. While GOLD ECOPD history categories are not intended to serve as a prognostic index of mortality, mortality is a major outcome of interest of COPD.^11,12,20^ This study showed that similar to ECOPD, GOLD’s current model had a limited discriminative accuracy to estimate 4-year all-cause mortality. Studying individual associations of prior moderate and severe ECOPD with mortality risk revealed that a history of 3 moderate ECOPD and 1 severe ECOPD event within 12 months were best at estimating 4-year all-cause mortality. Patients with 3 or more moderate ECOPD were more likely to die, with an OR of 2.18, whereas patients with previous severe ECOPD were more likely to die, with an OR of 1.57, within 4 years compared with patients without previous ECOPD. Thus, it required 3 or more moderate ECOPD to statistically observe the association of a severe ECOPD with all-cause mortality in this cohort.

### Strengths and Limitations

Strengths of this study were the large and well-characterized study population and the longitudinal follow-up period to study ECOPD and its trajectories for future COPD outcomes. However, several limitations should be noted. First, ECOPD data were collected during standardized interviews and relied on self-report. ECOPD were defined as acute and significant worsenings of lung disease that lasted several days and required taking special measures. It is noteworthy that these special measures were not specified and thus were susceptible to individual interpretation. In addition, a lack of exact timing of events precluded time-to-event analyses. Moreover, the 6-month time overlap between the first 2 visits (V1 and V2) and the 6-month time gap over subsequent visits (between V3 and V4 and V4 and V5) resulted in the cross-sectional approach of these analyses. Unfortunately, only 36% of patients had ECOPD data available at each study visit. However, these analyses were performed cross-sectionally, including all patients with available data at V2 and V3 for 1-year risk estimations, corresponding to 1687 and 1688 included patients with moderate and severe ECOPD, respectively, and at V2 and V5 for 4-year risk estimations, corresponding to 861 and 856 included patients with severe ECOPD, respectively. Because mortality rates were not available for each study visit, we could not associate ECOPD history with mortality. Nonetheless, ECOPD history trajectories of patients who did not survive were analyzed, and like survivors, most of these patients demonstrated a variable ECOPD history. Additionally, this study concerns a multicenter but single-country European cohort, which may raise concerns about the homogeneity and diversity of participants and thus the external validity of our findings.

## Conclusions

This cohort study, a multicenter secondary analysis of the German COSYCONET cohort, found that GOLD’s current ECOPD history categories were limited at estimating ECOPD and all-cause mortality risk but that lowering the threshold for moderate ECOPD was associated with improved performance. These findings suggest that patients may need to be referred to as having no exacerbations or having high-risk exacerbations. Importantly, a history of 1 or more moderate ECOPD was associated with a similar risk of future moderate ECOPD as a history of 1 or more severe ECOPD. Future studies are needed to validate the proposed cutoffs and elaborate further on other determinants aside from ECOPD history to estimate ECOPD risk, as well as their optimal, clinically applicable combination with the proposed novel approach. Moreover, the association of this lower cutoff with potential overtreatment of patients and subsequent financial implications needs to be evaluated.

## Notes

Supplement 1.
eMethods.

eResults.
**eFigure 1.** Timeline COSYCONET **eTable 1.** Baseline characteristics **eFigure 2.** Sankey diagrams **eFigure 3.** AUROC curve GOLD for moderate ECOPD risk **eFigure 4.** Precision recall curves GOLD for moderate ECOPD risk **eTable 2.** AUROC GOLD for moderate ECOPD risk (1-year estimation) **eTable 3.** AUROC GOLD for moderate ECOPD risk (4-year estimation) **eTable 4.** Contribution of covariates in adjusted odds ratios for moderate ECOPD status during study follow-up vs moderate and severe ECOPD history at V2 (1- and 4-year estimation) **eTable 5.** Contribution of covariates in adjusted odds ratios for severe ECOPD status during study follow-up vs moderate and severe ECOPD history at V2 (1- and 4-year estimation) **eFigure 5.** AUROC curve GOLD for severe ECOPD risk **eFigure 6.** Precision recall curves GOLD for severe ECOPD risk **eTable 6.** AUROC GOLD for severe ECOPD risk (1-year estimation) **eTable 7.** AUROC GOLD for severe ECOPD risk (4-year estimation) **eFigure 7.** AUROC curve GOLD to estimate 4-year all-cause mortality **eFigure 8.** Precision recall curve GOLD to estimate 4-year all-cause mortality **eTable 8.** AUROC GOLD to estimate 4-year all-cause mortality **eTable 9.** Odds ratios for all-cause mortality **eTable 10.** Contribution of covariates in adjusted odds ratios for all-cause mortality during study follow-up vs moderate and severe ECOPD history at V2 (4-year estimation) **eTable 11.** Paired-sample area difference under ROC curves **eFigure 9.** Precision recall curves GOLD and proposed ECOPD history grading for moderate and severe ECOPD risk
eReferences.

Supplement 2.
Data Sharing Statement
