# Supplementary material for: GOLD COPD Exacerbation History Categories and Disease Outcomes
Source: JAMA Netw Open. 2024 Dec 18;7(12):e2445488. doi: 10.1001/jamanetworkopen.2024.45488 (PMC11656261; doi:10.1001/jamanetworkopen.2024.45488)
Supplement: Supplement 1. — eMethods. eResults. eFigure 1. Timeline COSYCONET eTable 1. Baseline characteristics eFigure 2. Sankey diagrams eFigure 3. AUROC curve GOLD for moderate ECOPD risk eFigure 4. Precision recall curves GOLD for moderate ECOPD risk eTable 2. AUROC GOLD for moderate ECOPD risk (1-year estimation) eTable 3. AUROC GOLD for moderate ECOPD risk (4-year estimation) eTable 4. Contribution of covariates in adjusted odds ratios for moderate ECOPD status during study follow-up vs moderate and severe ECOPD history at V2 (1- and 4-year estimation) eTable 5. Contribution of covariates in adjusted odds ratios for severe ECOPD status during study follow-up vs moderate and severe ECOPD history at V2 (1- and 4-year estimation) eFigure 5. AUROC curve GOLD for severe ECOPD risk eFigure 6. Precision recall curves GOLD for severe ECOPD risk eTable 6. AUROC GOLD for severe ECOPD risk (1-year estimation) eTable 7. AUROC GOLD for severe ECOPD risk (4-year estimation) eFigure 7. AUROC curve GOLD to estimate 4-year all-cause mortality eFigure 8. Precision recall curve GOLD to estimate 4-year all-cause mortality eTable 8. AUROC GOLD to estimate 4-year all-cause mortality eTable 9. Odds ratios for all-cause mortality eTable 10. Contribution of covariates in adjusted odds ratios for all-cause mortality during study follow-up vs moderate and severe ECOPD history at V2 (4-year estimation) eTable 11. Paired-sample area difference under ROC curves eFigure 9. Precision recall curves GOLD and proposed ECOPD history grading for moderate and severe ECOPD risk eReferences. [file jamanetwopen-e2445488-s001.pdf]

Supplemental Online Content

Waeijen-Smit K, Peerlings DEM, Jörres RA, et al. GOLD COPD exacerbation history categories and disease outcomes. *JAMA Netw Open*. 2024;7(11):e2445488. doi:10.1001/jamanetworkopen.2024.45488

eMethods.

eResults.

eFigure 1. Timeline COSYCONET

eTable 1. Baseline characteristics

eFigure 2. Sankey diagrams

eFigure 3. AUROC curve GOLD for moderate ECOPD risk

eFigure 4. Precision recall curves GOLD for moderate ECOPD risk

eTable 2. AUROC GOLD for moderate ECOPD risk (1-year estimation)

eTable 3. AUROC GOLD for moderate ECOPD risk (4-year estimation)

eTable 4. Contribution of covariates in adjusted odds ratios for moderate ECOPD status during study follow-up vs moderate and severe ECOPD history at V2 (1- and 4-year estimation)

eTable 5. Contribution of covariates in adjusted odds ratios for severe ECOPD status during study follow-up vs moderate and severe ECOPD history at V2 (1- and 4-year estimation)

eFigure 5. AUROC curve GOLD for severe ECOPD risk

eFigure 6. Precision recall curves GOLD for severe ECOPD risk

eTable 6. AUROC GOLD for severe ECOPD risk (1-year estimation)

eTable 7. AUROC GOLD for severe ECOPD risk (4-year estimation)

eFigure 7. AUROC curve GOLD to estimate 4-year all-cause mortality

eFigure 8. Precision recall curve GOLD to estimate 4-year all-cause mortality

eTable 8. AUROC GOLD to estimate 4-year all-cause mortality

eTable 9. Odds ratios for all-cause mortality

eTable 10. Contribution of covariates in adjusted odds ratios for all-cause mortality during study follow-up vs moderate and severe ECOPD history at V2 (4-year estimation)

eTable 11. Paired-sample area difference under ROC curves

eFigure 9. Precision recall curves GOLD and proposed ECOPD history grading for moderate and severe ECOPD risk

eReferences.

This supplemental material has been provided by the authors to give readers additional information about their work.

© 2024 Waeijen-Smit K and Peerlings DEM et al. *JAMA Network Open.*

eMethods

The frequency of moderate and/or severe ECOPD in the last 12 months was collected during standardized interviews during the baseline visit (V1) and during the follow-up visits at 6- (V2), 18- (V3), 36- (V4) and 54-months (V5), as depicted in eFigure 1. It should be noted that, by default, an overlap of six months exists between the baseline visit and second visit, whilst a 6-month time gap exists between V3 and V4, and between V4 and V5.

Measurements

To study the risk of future moderate and severe ECOPD and all-cause mortality in relation to ECOPD history, the following baseline data were included: age (years), sex (male/female), time since diagnosis of COPD (years), GOLD group (ABE according to the GOLD 2023 report (1)), smoking status (never, current and former smoker), pack years, presence of cardiac comorbidities including heart failure, cardiac arrhythmia and cardiac arrest, oxygen therapy (yes/no) and COPD-related maintenance mono-, dual- and triple pharmacotherapy with long-acting β2-agonists (LABA), long-acting muscarinic antagonists (LAMA), and/or inhaled corticosteroids (ICS). Lung function assessments included the post-bronchodilator forced expiratory volume in 1 second (FEV_1) and the forced vital capacity (FVC). Cutoff FEV_1/FVC ratio for diagnosing COPD was <0.70 according to GOLD (2). Diffusing capacity was assessed using the lung transfer factor for carbon monoxide (TLCO). These procedures were performed in accordance with international guidelines and recommendations (3). Furthermore, the modified Medical Research Council (mMRC) scale (0-4) and the COPD assessment Test (CAT, 0-40) were also collected. mMRC scores were separated into less dyspnoeic (mMRC <2) and more dyspnoeic (≥2 mMRC). As cutoff value for CAT, 10 was used as proposed by GOLD recommendations.

Due to the occurrence of missing data of ECOPD history at some study visits, Sankey diagrams were constructed of the patients with ECOPD data available at each study visit only. Furthermore, since mortality rates were not available for each study visit, i.e. only for V4 and V5, mortality could not be integrated in the Sankey diagrams. ECOPD history trajectories of the non-surviving patients were therefore constructed separately.

eResults

In COSYCONET, a total of 2741 patients with a diagnosis of COPD participated in the baseline visit between September 2010 and December 2013. At the baseline visit, 450 patients exhibited a FEV_1/FVC ratio above 70%. Further details about this issue have previously been described elsewhere (3). These patients, formerly also described as GOLD 0, were excluded from the current post hoc analyses.

ECOPD history trajectories during study follow-up

eFigure 2A displays the flow and distribution of moderate ECOPD of the patients with data on moderate ECOPD available at each study visit. The majority of patients without ECOPD at V2 (56.9%) remained in the non-exacerbating group (69.9%), 20.6% experienced 1 moderate ECOPD, and 9.6% experienced ≥2 moderate ECOPD at the subsequent visit. The majority of patients with 1 previous ECOPD at V2 (22.8%) did not experience any ECOPD (39.7%), 31.2% experienced another single ECOPD, and 29.1% experienced ≥2 ECOPD at V3. Over one fourth of patients with ≥2 ECOPD at V2 (20.3%) did not experience any ECOPD (28.6%), 22.6% experienced 1 ECOPD, and 48.8% continued to experience ≥2 ECOPD at the subsequent visit. Similar patterns were observed during the remaining follow-up time. Overall, the vast majority of patients (79.6%) had a variable ECOPD history, 17.5% consistently remained in the non-exacerbating group, whereas 2.9% of patients consistently experienced ≥2 moderate ECOPD during study follow-up.

Since mortality could not be included in the Sankey diagram, ECOPD history trajectories of the non-surviving patients were explored separately (Figures not shown). Of the 219 non-surviving patients, 65 patients (29.7%) had complete ECOPD data available: 83.1% had a variable moderate ECOPD history, 13.8% consistently remained in the non-exacerbating group, and 1.5% consistently experienced ≥2 moderate ECOPD.

eFigure 2B displays the flow and distribution of severe ECOPD of the patients with data on severe ECOPD available at each study visit. The vast majority of patients without severe ECOPD at V2 (90.5%), continued to experience no severe ECOPD (90.5%), whereas 9.5% of patients experienced ≥1 severe ECOPD during the next year. More than half of the patients with ≥1 previous severe ECOPD at V2 (9.5%) did not experience any further severe ECOPD (57.7%), whilst 42.3% of patients continued to experience ≥1 severe ECOPD during the next year. Similar patterns were observed during the remaining follow-up time. In the end, the majority of patients (63.5%) consistently remained in the non(-severe) exacerbating group, whereas 0.8% of patients consistently experienced ≥1 severe ECOPD during study follow-up. Overall, 35.7% of patients had a variable severe ECOPD history.

Severe ECOPD history trajectories of the non-surviving patients were explored separately: 65 out of the 219 non-surviving patients (29.7%) had complete data on severe ECOPD history available at each study visit (Figures not

© 2024 Waeijen-Smit K and Peerlings DEM et al. *JAMA Network Open.*

shown). 38.5% of patients consistently remained in the non(-severe) exacerbating group, whereas 61.5% had a variable severe ECOPD history.

eFigures and eTables

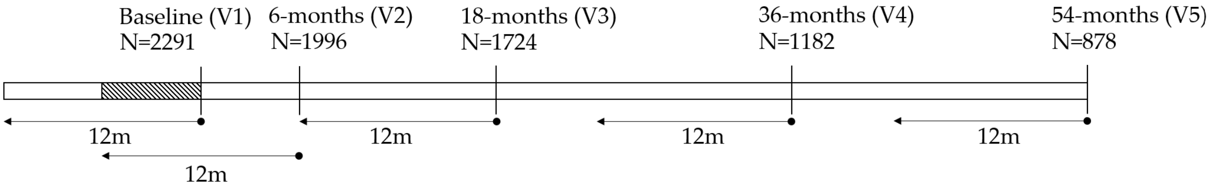

eFigure 1. Timeline COSYCONET. The five study visits, presented as V1-V5, were performed at baseline, 6-, 18-, 36- and 54-months, respectively. Exacerbation history <12 months was recorded at each visit (indicated by the arrows). The striped bar presents the 6-month overlap in exacerbation history between V1 and V2.

© 2024 Waeijen-Smit K and Peerlings DEM et al. *JAMA Network Open.*

eTable 1. Baseline characteristics of the 828 patients with ECOPD data available at each study visit.

	**Visit 1**<br>**N=828**
Sex,<br>Male<br>Female	 493 (59.5)<br>335 (40.5%)
Age	63.7±8.2
Years since diagnosis	6.0 (3.0-11.0)<br>N=824
GOLD A<br>GOLD B<br>GOLD E	116 (14.0)<br>456 (55.1)<br>255 (30.8)<br>N=827
FEV_1 % pred	57.7±18.0
FVC % pred	83.0±18.2
TLCO%	57.4±21.0<br>N=805
Current smoker<br>Ex-smoker<br>Never smoker	185 (22.3)<br>584 (70.5)<br>59 (7.1)
Pack years	41.0 (19.6-65.0)<br>N=823
LABA<br>LAMA<br>ICS<br>LABA/LAMA<br>ICS/LAMA<br>ICS/LABA<br>ICS/LABA/LAMA	48 (5.8)<br>58 (7.0)<br>9 (1.1)<br>120 (14.5)<br>13 (1.6)<br>102 (12.3)<br>397 (47.9)
Oxygen use, yes	106 (12.8)
mMRC ≥2	311 (37.7)<br>N=825
CAT ≥10	702 (85.1)<br>N=825
Number of moderate ECOPD <12 months<br>0<br>1<br>2<br>3<br>≥4	 <br>414 (50.0)<br>214 (25.8)<br>97 (11.7)<br>40 (4.8)<br>63 (7.6)
Number of severe ECOPD <12 months<br>0<br>1<br>2<br>3<br>≥4	 <br>706 (85.3)<br>92 (11.1)<br>22 (2.7)<br>5 (0.6)<br>3 (0.4)
Heart failure, yes	39 (9.4)<br>N=415
Cardiac arrhythmia, yes	59 (14.2)<br>N=416
Cardiac arrest, yes	29 (3.5)

Variables are presented as: n (%), mean ± standard deviation, median (interquartile range). In case of missing data, the total number of patients for which the variable was available is presented (N).

© 2024 Waeijen-Smit K and Peerlings DEM et al. *JAMA Network Open.*

A Sankey Diagram of moderate ECOPD during study follow-up

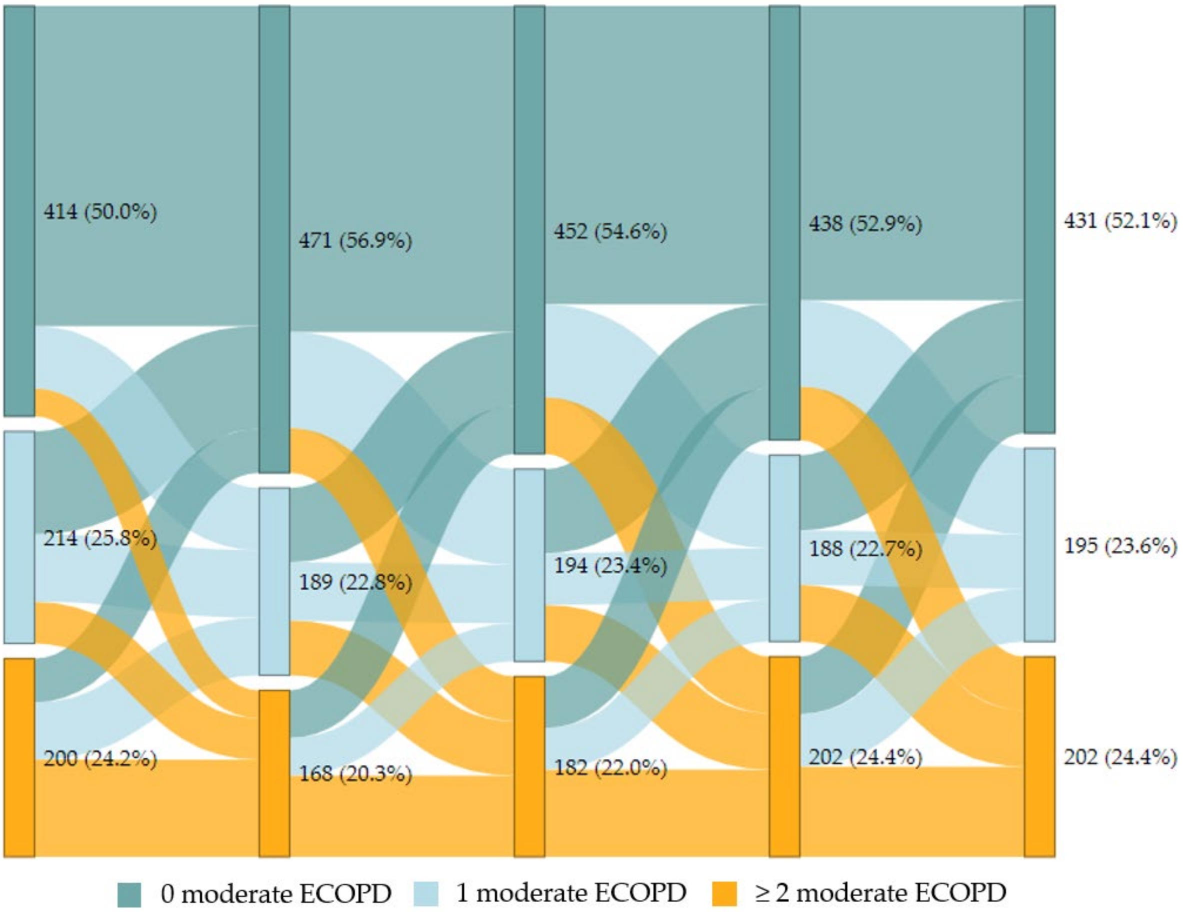

© 2024 Waeijen-Smit K and Peerlings DEM et al. *JAMA Network Open.*

B Sankey Diagram of severe ECOPD during study follow-up

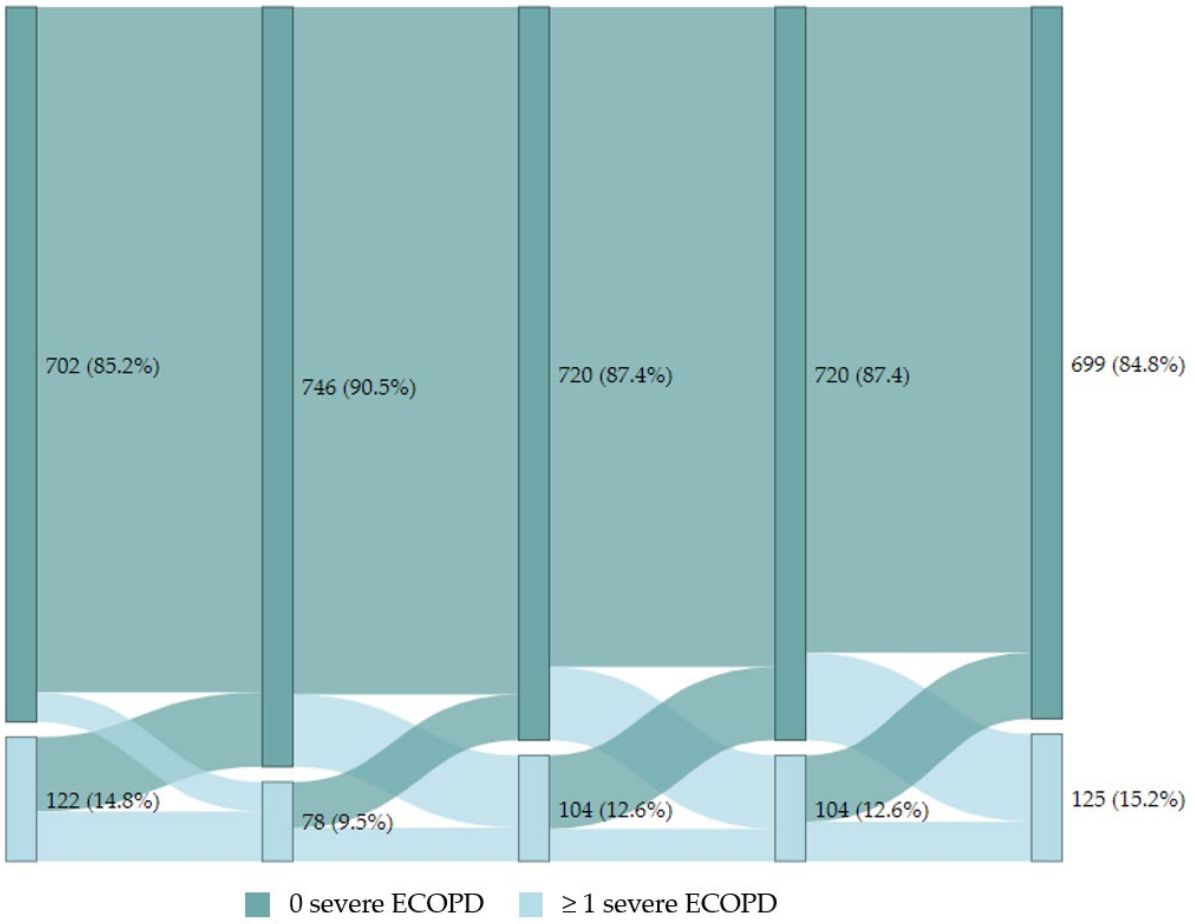

eFigure 2. Sankey diagram displaying the flow and distribution of the different frequencies of A: moderate (n=828) and B: severe (n=824) ECOPD during study follow-up.

© 2024 Waeijen-Smit K and Peerlings DEM et al. *JAMA Network Open.*

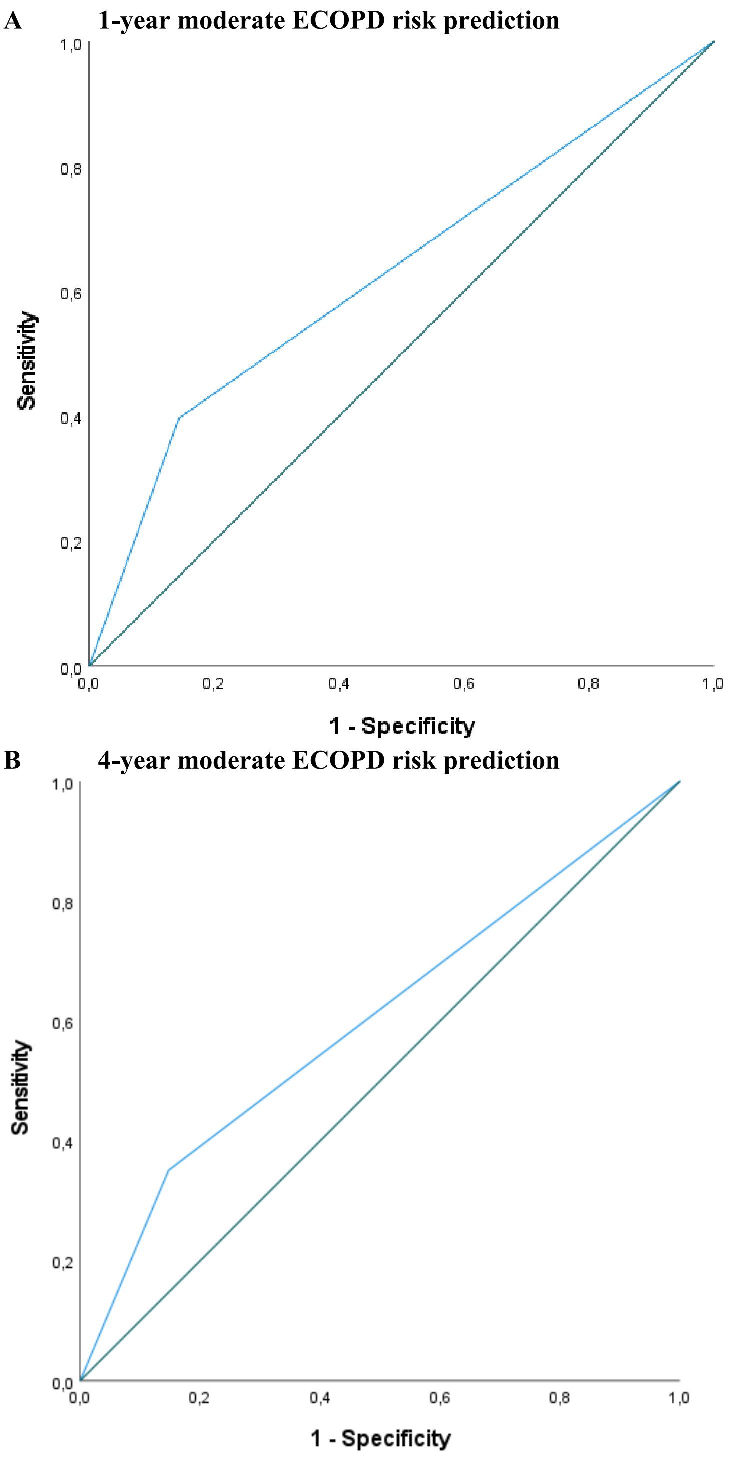

eFigure 3. ROC curve of the ECOPD history categories by GOLD to predict A: 1-year (V2 vs. V3, n=1687) and B: 4-year (V2 vs. V5, n=861) moderate ECOPD risk. Green; reference line.

© 2024 Waeijen-Smit K and Peerlings DEM et al. *JAMA Network Open.*

eTable 2. AUC with 95% CI and individual sensitivity and specificity scores per cutoff value (V2) for 1-year moderate ECOPD risk prediction (V3).

	**Sensitivity**	**Specificity**	**Youden's index**	**AUC (95% CI)**
0 moderate ECOPD <12 months	Ref	Ref	Ref	0.68 (0.65, 0.70)
1 moderate ECOPD <12 months	62.4%	70.1%	**0.324**	
2 moderate ECOPD <12 months	32.4%	89.2%	0.217	
3 moderate ECOPD <12 months	19.0%	94.7%	0.137	
≥4 moderate ECOPD <12 months	10.1%	97.2%	0.072	
0 severe ECOPD <12 months	Ref	Ref	Ref	0.55 (0.53, 0.58)
1 severe ECOPD <12 months	17.1%	93.4%	**0.106**	
2 severe ECOPD <12 months	5.0%	98.4%	0.034	
3 severe ECOPD <12 months	2.2%	99.5%	0.018	
≥4 severe ECOPD <12 months	0.09%	99.8%	0.006	

eTable 3. AUC with 95% CI and individual sensitivity and specificity scores per cutoff value (V2) for 4-year moderate ECOPD risk prediction (V5).

	**Sensitivity**	**Specificity**	**Youden's index**	**AUC (95% CI)**
0 moderate ECOPD <12 months	Ref	Ref	Ref	0.64 (0.60, 0.68)
1 moderate ECOPD <12 months	55.7%	68.1%	**0.238**	
2 moderate ECOPD <12 months	30.3%	88.8%	0.191	
3 moderate ECOPD <12 months	18.2%	95.1%	0.132	
≥4 moderate ECOPD <12 months	9.7%	98.0%	0.077	
0 severe ECOPD <12 months	Ref	Ref	Ref	0.54 (0.50, 0.58)
1 severe ECOPD <12 months	14.0%	94.4%	**0.085**	
2 severe ECOPD <12 months	4.1%	98.9%	0.030	
3 severe ECOPD <12 months	1.2%	99.6%	0.008	
≥4 severe ECOPD <12 months	0.5%	100%	0.005	

© 2024 Waeijen-Smit K and Peerlings DEM et al. *JAMA Network Open.*

eTable 4: Contribution of the covariates in the adjusted odds ratios for moderate ECOPD status during study follow-up versus moderate and severe ECOPD history (1- and 4-year estimation).

	**1-year moderate ECOPD OR (95% CI)**	**4-year moderate ECOPD OR (95% CI)**
0 Moderate ECOPD <12 months	**Ref**	**Ref**
≥1 Moderate ECOPD <12 months	**1.71 (1.52-1.92, *Sig.*<0.001**	**1.65 (1.40-1.95), *Sig.*<0.001**
- Age (years)	**0.99 (0.98-1.01), *Sig.*=0.205**	**0.99 (0.97-1.01), *Sig.*=0.243**
- Sex (males vs. females)	**1.12 (0.87-1.44), *Sig.*=0.389**	**1.18 (0.83-1.69), *Sig.*=0.364**
- FEV1 (% pred)	**0.98 (0.98-0.99), *Sig.*<0.001**	**0.97 (0.96-0.98), *Sig.*<0.001**
- Smoking status (current vs. never smoker)	**0.78 (0.47-1.31), *Sig.*=0.349**	**0.87 (0.42-1.80), *Sig.*=0.714**
- Cardiac arrhythmia (yes vs. no)	**1.28 (0.87-1.89), *Sig.* =2.15**	**2.61 (1.44-4.73), *Sig.*=0.002**
- Heart failure (yes vs. no)	**1.19 (0.75-1.89), *Sig.* =0.458**	**1.14 (0.57-2.28), *Sig.*=0.716**
- Cardiac Arrest (yes vs. no)	**0.85 (0.46-1.56), *Sig.* =0.596**	**0.98 (0.36-2.60), *Sig.*=0.947**
0 Severe ECOPD<12 months	**Ref**	**Ref**
≥1 Severe ECOPD<12 months	**1.84 (1.42-2.39), *Sig.*<0.001**	**1.77 (1.18-2.65), *Sig.*=0.006**
- Age (years)	**0.98 (0.97-1.00), *Sig.* =0.037**	**0.98 (0.96-1.01), *Sig.*=0.136**
- Sex (males vs. females)	**1.15 (0.90-1.46), *Sig.* =0.259**	**1.23 (0.87-1.74), *Sig.*=0.235**
- FEV1 (% pred)	**0.98 (0.97-0.99), *Sig.* <0.001**	**0.97 (0.96-0.98), *Sig.*<0.001**
- Smoking status (current vs. never smoker)	**0.84 (0.67-107), *Sig.* =0.154**	**0.73 (0.37-1.47), *Sig.*=0.382**
- Cardiac arrhythmia (yes vs. no)	**1.7 (0.94-1.99), *Sig.* =0.100**	**2.54 (1.43-4.51), *Sig.*=0.002**
- Heart failure (yes vs. no)	**1.18 (0.76-1.85), *Sig.* =0.463**	**1.15 (0.58-2.27), *Sig.*=0.689**
- Cardiac Arrest (yes vs. no)	**0.86 (0.48-1.55), *Sig.* =0.614**	**0.95 (0.36-2.58), *Sig.*=0.919**

eTable 5: Contribution of the covariates in the adjusted odds ratios for severe ECOPD status during study follow-up versus moderate and severe ECOPD history (1- and 4-year estimation).

	**1-year severe ECOPD OR (95% CI)**	**4-year severe ECOPD OR (95% CI)**
0 Moderate ECOPD <12 months	**Ref**	**Ref**
≥1 Moderate ECOPD <12 months	**1.28 (1.13-1.46), *Sig.*<0.001**	**1.29 (1.08-1.55), *Sig.*=0.005**
- Age (years)	**1.03 (1.00-1.05), *Sig.*=0.023**	**1.04 (1.01-1.08), *Sig.*=0.007**
- Sex (males vs. females)	**1.12 (0.79-1.59), *Sig.*=0.513**	**1.11 (0.69-1.78), *Sig.*=0.679**
- FEV1 (% pred)	**0.97 (0.96-0.98), *Sig.*<0.001**	**0.95 (0.94-0.97), *Sig.*<0.001**
- Smoking status (current vs. never smoker)	**1.09 (0.54-2.22), *Sig.*=0.802**	**0.81 (0.34-1.94), *Sig.*=0.643**
- Cardiac arrhythmia (yes vs. no)	**1.38 (0.85-2.24), *Sig.*=0.197**	**1.64 (0.82-3.30), *Sig.*=0.162**
- Heart failure (yes vs. no)	**1.56 (0.90-2.71), *Sig.*=0.117**	**1.17 (0.50-2.72), *Sig.*=0.721**
- Cardiac Arrest (yes vs. no)	**1.27 (0.59-2.76), *Sig.*=0.540**	**1.32 (0.39-4.44), *Sig.*=0.658**
0 Severe ECOPD<12 months	**Ref**	**Ref**
≥1 Severe ECOPD<12 months	**2.30 (1.80-2.94), *Sig.*<0.001**	**2.35 (0.42-4.79), *Sig.*<0.001**
- Age (years)	**1.02 (1.00-1.04), *Sig.*=0.132**	**1.04 (1.01-1.07), *Sig.*=0.018**
- Sex (males vs. females)	**1.12 (0.78-1.59), *Sig.*=0.541**	**1.10 (0.68-1.78), *Sig.*=0.699**
- FEV1 (% pred)	**0.97 (0.96-0.98), *Sig.*<0.001**	**0.96 (0.94-0.97), *Sig.*<0.001**
- Smoking status (current vs. never smoker)	**1.00 (0.49-2.04), *Sig.*=0.995**	**0.75 (0.32-1.81), *Sig.*=0.527**
- Cardiac arrhythmia (yes vs. no)	**1.39 (0.85-2.28), *Sig.*=0.194**	**1.52 (0.74-3.10), *Sig.*=0.256**
- Heart failure (yes vs. no)	**1.61 (0.91-2.84), *Sig.*=0.101**	**1.25 (0.53-2.95), *Sig.*=0.611**
- Cardiac Arrest (yes vs. no)	**1.13 (0.50-2.54), *Sig.*=0.773**	**1.20 (0.34-4.23), *Sig.*=0.777**

© 2024 Waeijen-Smit K and Peerlings DEM et al. *JAMA Network Open.*

A 1-year moderate ECOPD risk prediction

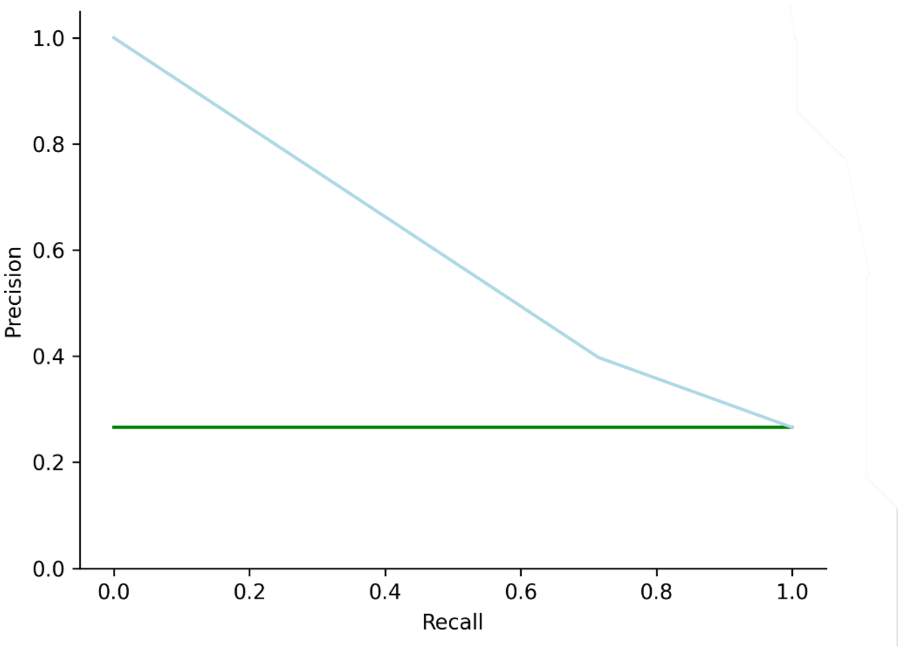

B 4-year moderate ECOPD risk prediction

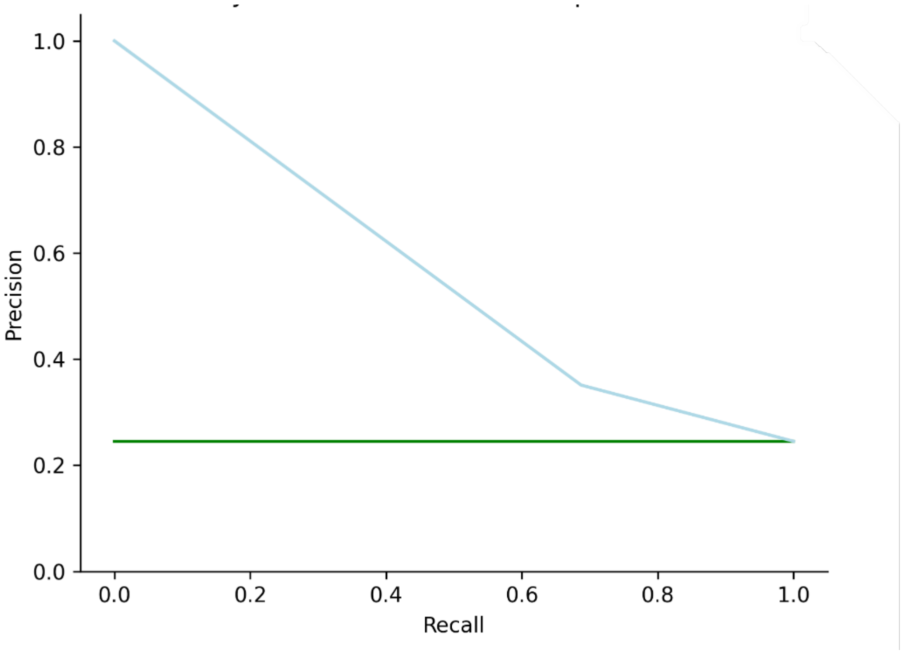

eFigure 4. Precision Recall curves of the ECOPD history categories by GOLD to predict A: 1-year (V2 vs. V3, n=1687; f1=0.000, AUC=0.594) and B: 4-year (V2 vs. V5, n=861; f1=0.000, AUC=0.557) moderate ECOPD risk. Green; reference line.

© 2024 Waeijen-Smit K and Peerlings DEM et al. *JAMA Network Open.*

A **1-year severe ECOPD risk prediction**

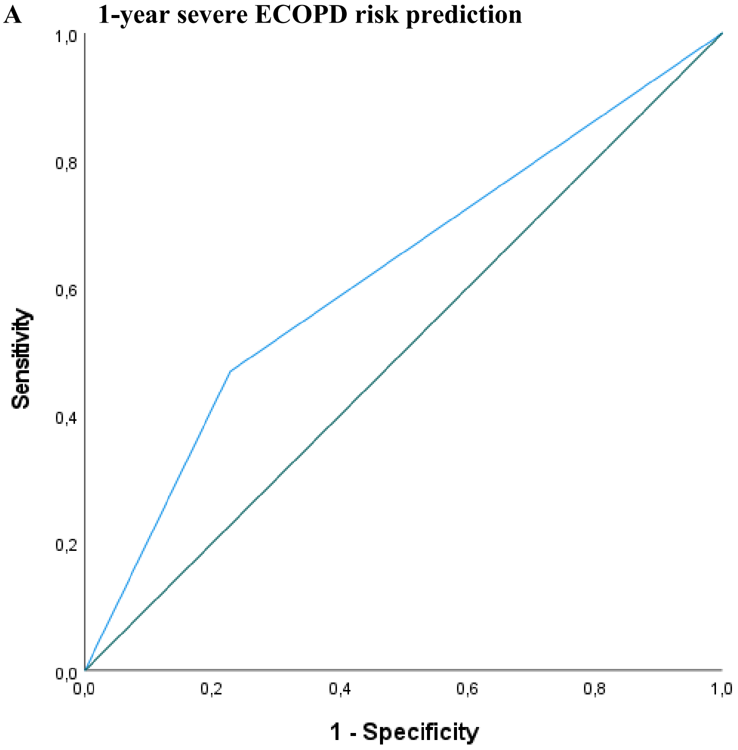

B **4-year severe ECOPD risk prediction**

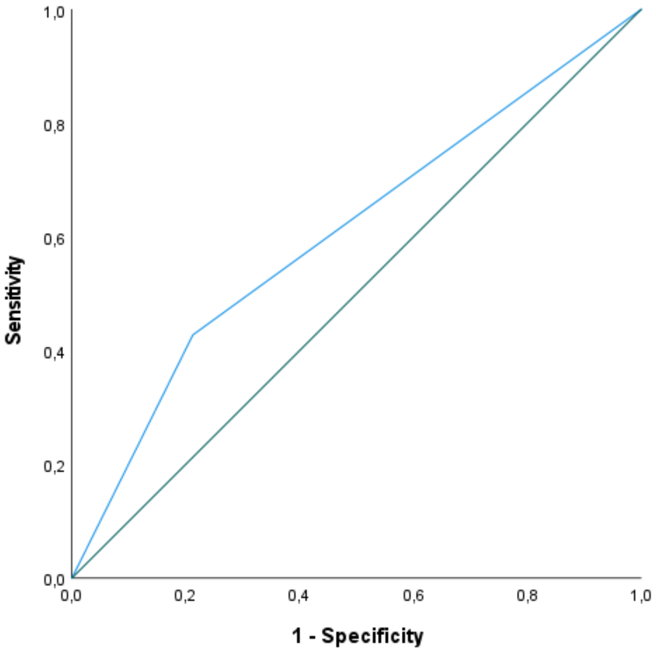

eFigure 5. ROC curve of the ECOPD history categories by GOLD to predict A: 1-year (V2 vs. V3, n=1688) and B: 4-year (V2 vs. V5, n=856) severe ECOPD risk . Green; reference line.

© 2024 Waeijen-Smit K and Peerlings DEM et al. *JAMA Network Open.*

eTable 6. AUC with 95% CI and individual sensitivity and specificity scores per cutoff value (V2) for 1-year severe ECOPD risk prediction (V3).

	**Sensitivity**	**Specificity**	**Youden's index**	**AUC (95% CI)**
0 moderate ECOPD <12 months	Ref	Ref	Ref	0.64 (0.60, 0.67)
1 moderate ECOPD <12 months	67.9%	58.2%	**0.262**	
2 moderate ECOPD <12 months	34.6%	81.1%	0.157	
3 moderate ECOPD <12 months	17.7%	89.1%	0.068	
≥4 moderate ECOPD <12 months	7.6%	93.9%	0.015	
0 severe ECOPD <12 months	Ref	Ref	Ref	0.62 (0.57, 0.66)
1 severe ECOPD <12 months	31.2%	91.6%	**0.228**	
2 severe ECOPD <12 months	9.3%	97.8%	0.071	
3 severe ECOPD <12 months	5.1%	99.3%	0.044	
≥4 severe ECOPD <12 months	1.7%	99.7%	0.013	

eTable 7. AUC with 95% CI and individual sensitivity and specificity scores per cutoff value (V2) for 4-year severe ECOPD risk prediction (V5).

	**Sensitivity**	**Specificity**	**Youden's index**	**AUC (95% CI)**
0 moderate ECOPD <12 months	Ref	Ref	Ref	0.61 (0.56, 0.66)
1 moderate ECOPD <12 months	59.5%	59.6%	**0.191**	
2 moderate ECOPD <12 months	32.8%	81.8%	0.146	
3 moderate ECOPD <12 months	19.1%	90.1%	0.092	
≥4 moderate ECOPD <12 months	9.9%	95.0%	0.050	
0 severe ECOPD <12 months	Ref	Ref	Ref	0.60 (0.54, 0.66)
1 severe ECOPD <12 months	26.0%	93.4%	**0.193**	
2 severe ECOPD <12 months	8.4%	98.5%	0.069	
3 severe ECOPD <12 months	2.3%	99.4%	0.017	
≥4 severe ECOPD <12 months	1.5%	100.0%	0.015	

© 2024 Waeijen-Smit K and Peerlings DEM et al. *JAMA Network Open.*

A **1-year severe ECOPD risk prediction**

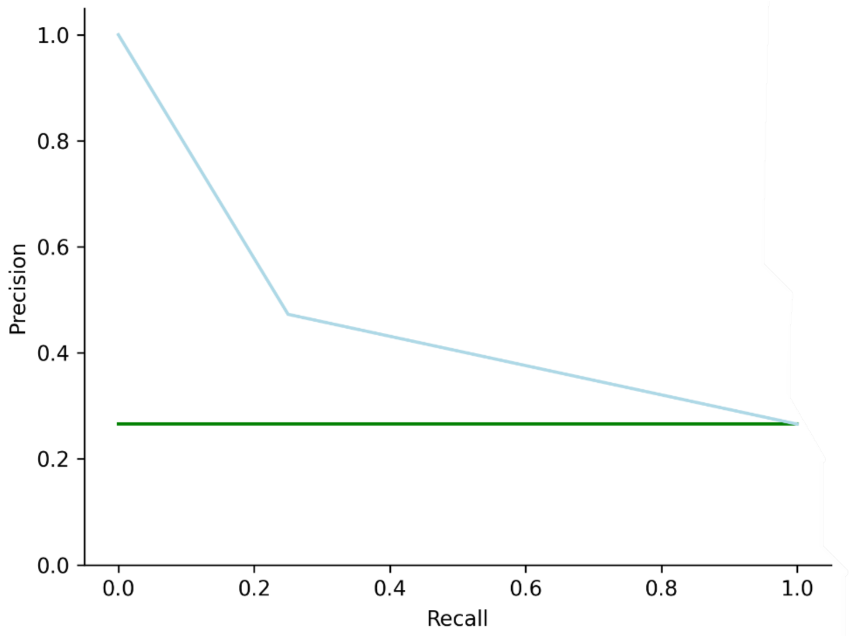

B **4-year severe ECOPD risk prediction**

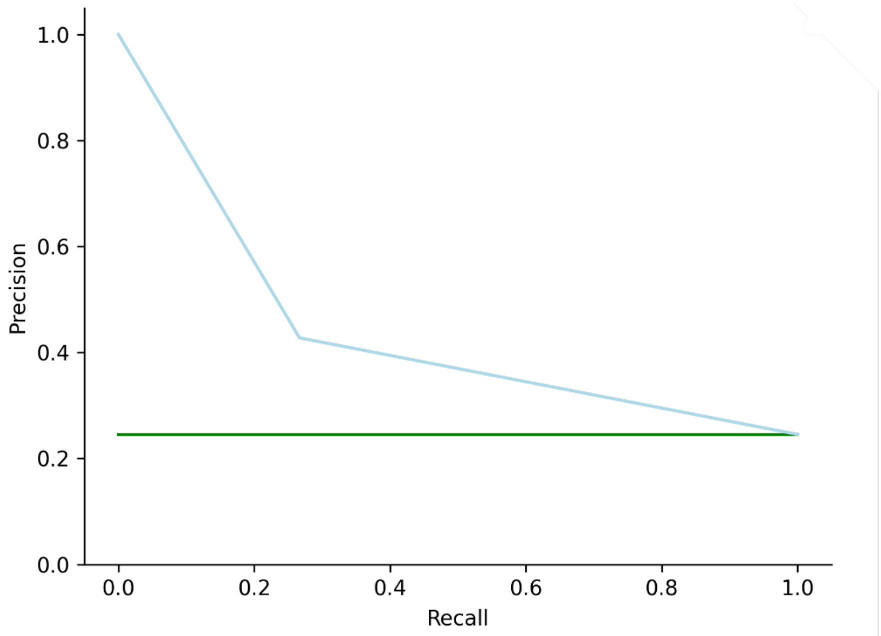

eFigure 6. Precision Recall curves of the ECOPD history categories by GOLD to predict A: 1-year (V2 vs. V3, n=1688; f1=0.000, AUC=0.461) and B: 4-year (V2 vs. V5, n=856; f=0.000, AUC=0.437) severe ECOPD risk . Green; reference line.

© 2024 Waeijen-Smit K and Peerlings DEM et al. *JAMA Network Open.*

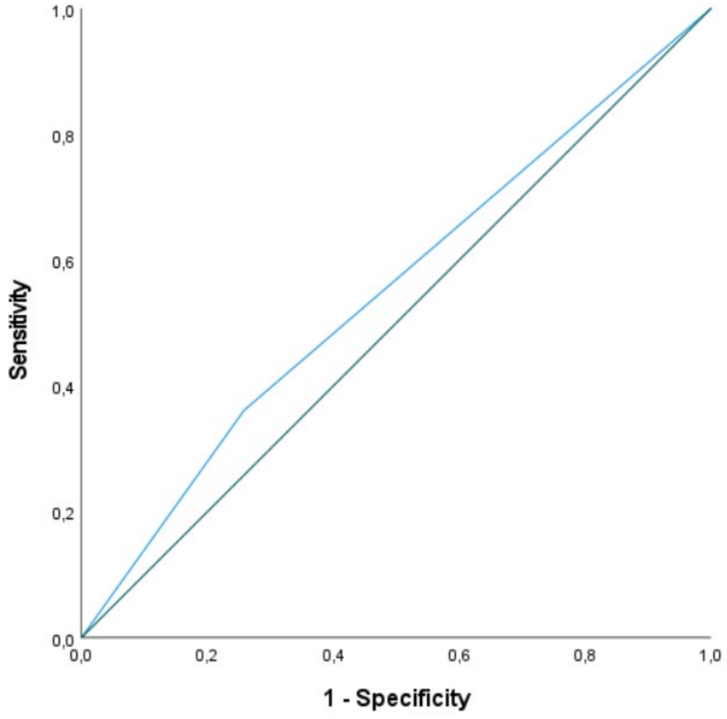

eFigure 7. ROC curve of the ECOPD history categories by GOLD to predict 4-year all-cause mortality (V2 vs. V5), n=1994. Green; reference line.

© 2024 Waeijen-Smit K and Peerlings DEM et al. *JAMA Network Open.*

eTable 8. AUC with 95% CI and individual sensitivity and specificity scores per cutoff value (V2) for 4-year all-cause mortality prediction (V5).

	**Sensitivity**	**Specificity**	**Youden's index**	**AUC (95% CI)**
0 moderate ECOPD <12 months	Ref	Ref	Ref	0.53 (0.49, 0.58)
1 moderate ECOPD <12 months	49.7%	54.7%	0.044	
2 moderate ECOPD <12 months	26.2%	79.0%	0.052	
3 moderate ECOPD <12 months	16.9%	88.5%	**0.054**	
≥4 moderate ECOPD <12 months	6.6%	93.6%	0.002	
0 severe ECOPD <12 months	Ref	Ref	Ref	0.57 (0.52, 0.62)
1 severe ECOPD <12 months	25.1%	88.9%	**0.140**	
2 severe ECOPD <12 months	6.6%	96.9%	0.035	
3 severe ECOPD <12 months	3.8%	98.8%	0.026	
≥4 severe ECOPD <12 months	0.5%	99.4%	0.000	

© 2024 Waeijen-Smit K and Peerlings DEM et al. *JAMA Network Open.*

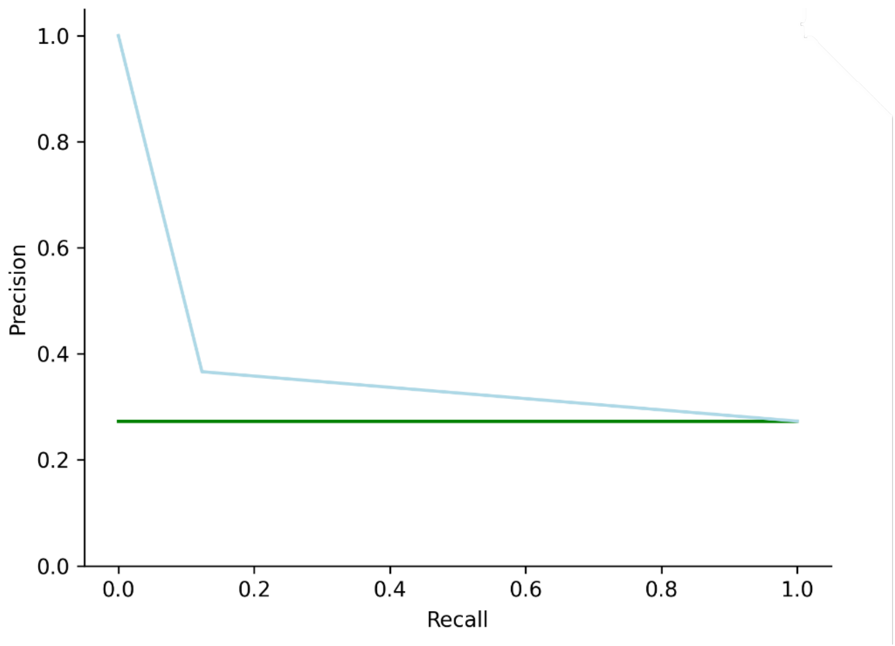

eFigure 8. Precision recall curve of the ECOPD history categories by GOLD to predict 4-year all-cause mortality (V2 vs. V5, n=1994; f1=0.000, AUC=0.364). Green; reference line.

© 2024 Waeijen-Smit K and Peerlings DEM et al. *JAMA Network Open.*

eTable 9. Crude and adjusted odds ratios for 54-month mortality, versus moderate and severe ECOPD history at V2.

	**Mortality at V5**	
	**Crude**	**Adjusted**
	**OR (95% CI)** ***Sig.***	**OR (95% CI)** ***Sig.***
0 moderate ECOPD <12 months	Ref	Ref
1 moderate ECOPD <12 months	1.05 (0.72-1.54) *0.795*	1.06 (0.66-1.70) *0.816*
2 moderate ECOPD <12 months	1.06 (0.62-1.83) *0.824*	1.20 (0.63-2.27) *0.583*
≥3 moderate ECOPD <12 months	2.18 (1.27-3.72) *0.005*	2.50 (1.30-4.80) *0.006*
X^2 (DF) *Sig*	7.16 (4) *0.128*	90.22 (11) *<0.001*
Nagelkerke R^2	0.8	13.6
N included in the analysis (%)	1994 (87.0%)	1425 (62.2%)
0 severe ECOPD <12 months	Ref	Ref
≥1 severe ECOPD <12 months	1.57 (1.29-1.91) *<0.001*	1.47 (1.14-1.90) *0.003*
X^2 (DF) *Sig*	16.66 (1) *<0.001*	90.99 (8) *<0.001*
Nagelkerke R^2	1.8	13.7
N included in the analysis (%)	1994 (87.0%)	1425 (62.2%)

Ref; reference. Adjustments for age, sex, smoking status, FEV_1 % predicted, and cardiac comorbidities (i.e. heart failure, cardiac arrhythmia and cardiac arrest) were performed.

© 2024 Waeijen-Smit K and Peerlings DEM et al. *JAMA Network Open.*

eTable 10: Contribution of the covariates in the adjusted odds ratios for all-cause mortality during study follow-up versus moderate and severe ECOPD history (4-year estimation).

	**Mortality at V5**<br>**OR (95% CI)**
0 Moderate ECOPD <12 months	**Ref**
1 Moderate ECOPD <12 months	**1.06 (0.66-1.70), *Sig.*=0.816**
2 Moderate ECOPD <12months	**1.20 (0.63-2.27), *Sig.*=0.582**
≥3 Moderate ECOPD <12 months	**2.50 (1.30-4.80), *Sig.*=0.006**
- Age (years)	**1.09 (1.06-1.12), *Sig.*<0.001**
- Sex (males vs. females)	**0.65 (0.43-1.00), *Sig.*=0.051**
- FEV1 (% pred)	**0.97 (0.96-0.98), *Sig.*<0.001**
- Smoking status (current vs. never smoker)	**3.64 (1.30-10.18), *Sig.*=0.014**
- Cardiac arrhythmia (yes vs. no)	**0.71 (0.40-1.27), *Sig.*=0.252**
- Heart failure (yes vs. no)	**2.30 (1.29-4.10), *Sig.*=0.005**
- Cardiac Arrest (yes vs. no)	**0.84 (0.33-2.12), *Sig.*=0.713**
0 Severe ECOPD<12 months	**Ref**
≥1 Severe ECOPD<12 months	**1.47 (1.14-1.90), *Sig.*=0.003**
- Age (years)	**1.08 (1.06-1.11), *Sig.*<0.001**
- Sex (males vs. females)	**0.65 (0.42-0.99), *Sig.*=0.044**
- FEV1 (% pred)	**0.97 (0.96-0.99), *Sig.*<0.001**
- Smoking status (current vs. never smoker)	**3.61 (1.29-10.12), *Sig.*=0.015**
- Cardiac arrhythmia (yes vs. no)	**0.72 (0.40-1.28), *Sig.*=0.257**
- Heart failure (yes vs. no)	**2.39 (1.34-4.27), *Sig.*=0.003**
- Cardiac Arrest (yes vs. no)	**0.83 (0.33-2.09), *Sig.*=0.691**

eTable 11. Paired-sample area difference under the ROC curves.

**Test**	**Z**	**Sig.**	**AUC difference**	**Std. error difference**	**95% CI**
Current GOLD grading vs. proposal for novel grading for 1-year moderate ECOPD risk	-3.881	<0.001	-0.037	0.148	-0.056, -0.018
Current GOLD grading vs. proposal for novel grading for 4-year moderate ECOPD risk	-1.183	0.237	-0.16	0.175	-0.042, 0.010
Current GOLD grading vs. proposal for novel grading for 1-year severe ECOPD risk	-0.850	0.395	-0.012	0.182	-0.040, 0.016
Current GOLD grading vs. proposal for novel grading for 4-year severe ECOPD risk	0.701	0.484	0.013	0.214	-0.023, 0.048

Sig; significance.

© 2024 Waeijen-Smit K and Peerlings DEM et al. *JAMA Network Open.*

A **1-year moderate ECOPD risk prediction**

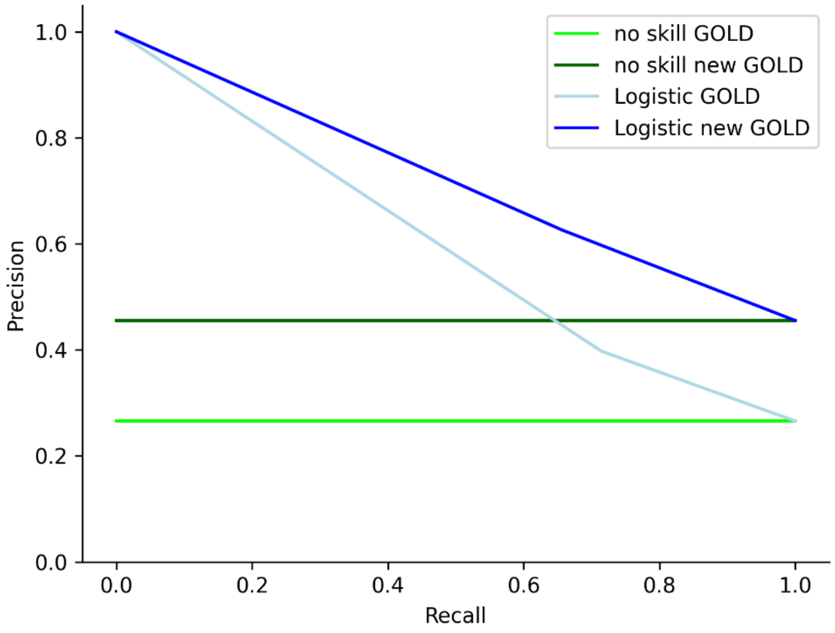

B **4-year moderate ECOPD risk prediction**

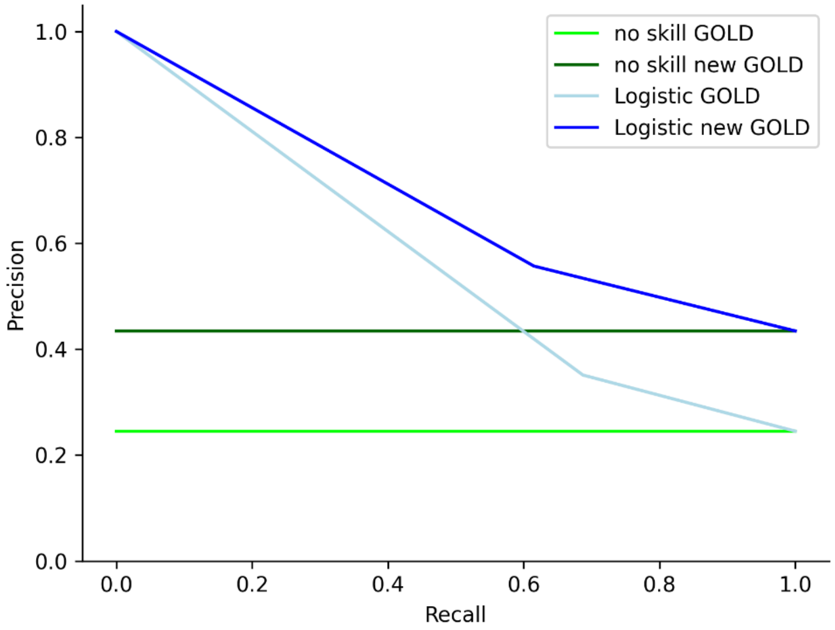

© 2024 Waeijen-Smit K and Peerlings DEM et al. *JAMA Network Open.*

C 1-year severe ECOPD risk prediction

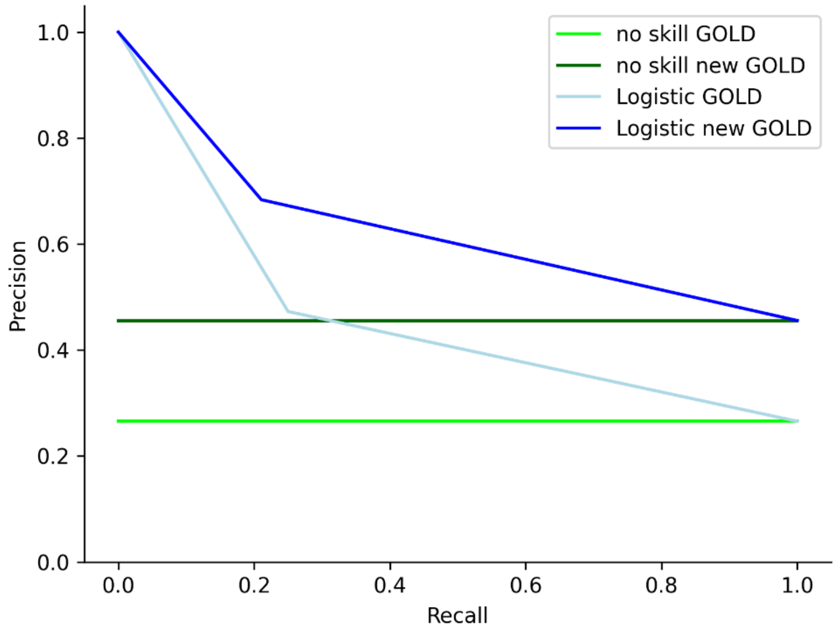

D 4-year severe ECOPD risk prediction

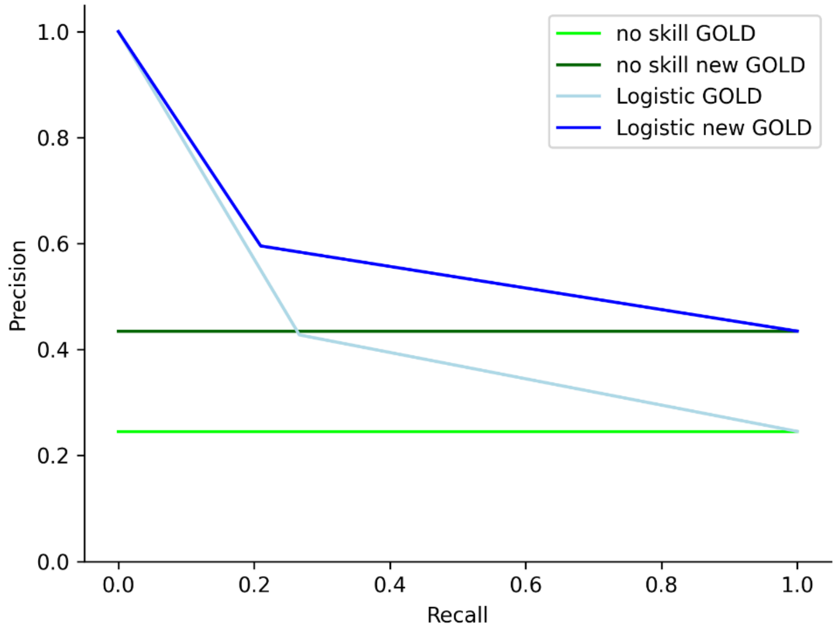

eFigure 9. Precision Recall curves of the ECOPD history categories by GOLD (light blue) and proposal for novel ECOPD history grading (dark blue) to estimate A: 1-year (V2 vs. V3, n = 1687) and B: 4-year (V2 vs. V5, n= 861) moderate ECOPD risk, and to estimate C: 1 year (V2 vs. V3, n=1688) and D: 4 year (V2 vs. V5, n=856 severe ECOPD risk. Light green: GOLD reference line, dark green: proposal for novel ECOPD history grading reference line.

eReferences

1. Global Initiative for Chronic Obstructive Lung Disease. Global strategy for the diagnosis, management, and prevention of chronic obstructive pulmonary disease. 2023 report. https://goldcopdorg/2023-gold-report-2/.
2. Global Initiative for Chronic Obstructive Lung Disease. Global strategy for the diagnosis, management, and prevention of chronic obstructive pulmonary disease. 2024 report. https://goldcopdorg/2024-gold-report/.

© 2024 Waeijen-Smit K and Peerlings DEM et al. *JAMA Network Open.*

3. Karch A, Vogelmeier C, Welte T, Bals R, Kauczor H-U, Biederer J, et al. The German COPD cohort COSYCONET: aims, methods and descriptive analysis of the study population at baseline. Respiratory medicine. 2016;114:27-37.

© 2024 Waeijen-Smit K and Peerlings DEM et al. *JAMA Network Open.*
