# Supplementary material for: GOLD COPD Exacerbation History Categories and Disease Outcomes
Source: JAMA Netw Open. 2024 Dec 18;7(12):e2445488. doi: 10.1001/jamanetworkopen.2024.45488 (PMC11656261; doi:10.1001/jamanetworkopen.2024.45488)
Supplement: Supplement 2. — Data Sharing Statement [file jamanetwopen-e2445488-s002.pdf]

Data Sharing Statement

Waeijen-Smit and Peerlings. GOLD COPD Exacerbation History Categories and Disease Outcomes. *JAMA Netw Open*. Published online November 19, 2024. doi:10.1001/jamanetworkopen.2024.45488

Data
Data available: No

Additional Information
Explanation for why data not available: Data access proposals can be send to the COSYCONET website.
